# Risk of SARS-CoV-2 infection in dental healthcare workers – a systematic review and meta-analysis

**DOI:** 10.3205/dgkh000464

**Published:** 2024-03-05

**Authors:** Kira Marie Schwarz, Albert Nienhaus, Roland Diel

**Affiliations:** 1 Institute for Health Service Research in Dermatology and Nursing (IVDP), University Medical Center Hamburg-Eppendorf, Hamburg, Germany; 2 Institution for Statutory Accident Insurance and Prevention in the Health and Welfare Services (BGW), Hamburg, Germany; 3 Institute for Epidemiology, University Medical Hospital, Schleswig-Holstein, Kiel, Germany; 4 LungClinic Grosshansdorf, Airway Research Center North (ARCN), German Center for Lung Research (DZL), Grosshansdorf, Germany

**Keywords:** COVID-19, SARS-CoV-2, infection risk, prevalence, incidence, personal protective equipment

## Abstract

**Background:**

Mounting evidence supports an association between the use of personal protective equipment (PPE) and the risk of infection from the severe acute respiratory syndrome coronavirus 2 (SARS-CoV-2) in dental healthcare workers (DCW). However, the prevalence and incidence of SARS-CoV-2 infections in the setting of dental care remains poorly characterized.

**Methods:**

A systematic review and meta-analysis of studies published prior to Mai 2023 providing epidemiological data for the occurrence of SARS-CoV-2 in DCW was performed. A random-effects model was used to calculate pooled estimates and odds ratios (ORs) with corresponding 95% confidence intervals (CIs). The associated factors were narratively evaluated. Risk of bias was assessed using the Joanna Briggs Institute tool for prevalence studies.

**Results:**

Twenty-nine eligible studies were identified including a total of 85,274 DCW at risk; 27 studies met the criteria for the meta-analysis. Among the included DCW, the overall prevalence of SARS-CoV-2 was 11.8% (13,155/85,274; 95%CI, 7.5%–17%), whereby the degree of heterogeneity between the studies was considerable (I^2^=99.7%). The pooled prevalence rate for dentists and dental hygienists alone was 12.7% (1943/20,860; 95%CI, 8.0%–18.0%), showing significantly increased odds of contracting a SARS-CoV-2 infection compared to dental assistant personnel, the prevalence rate for which was less than half, at 5.2% (613/15,066; OR=2.42; 95% CI, 2.2–2.7). In the subgroup of 17 studies from countries with high income there was a significantly lower prevalence rate of 7.3% (95% CI, 5%–10%) in DCW compared to the prevalence rate in low- and middle-income countries, which came to 20.8% (95% CI, 14%–29%; p<0.001). In 19 out of the 29 studies (65.5%), specific information on the use of and adherence to PPE was absent while in the reports with concrete figures the wearing of N95 (or at least surgical masks) by DCW appeared to be associated with lower SARS-CoV-2 prevalence rates.

**Conclusions:**

DCW were, depending in each case on their proximity to patients, at particular risk of SARS-CoV-2 infection during the COVID-19 pandemic. Until a significant level of vaccination protection against newer SARS-CoV-2 variants can be built up in the population, dental healthcare facilities should further maintain their focus on using PPE according to current guidelines.

## Zusammenfassung

**Hintergrund:**

Es besteht eine zunehmende Evidenz über den Zusammenhang zwischen dem Infektionsrisiko von zahnmedizinischem Personal (ZP) gegenüber SARS-CoV-2 und der Verwendung einer persönlichen Schutzausrüstung (PPE). Bislang sind Prävalenz und Inzidenz von SARS-CoV-2-Infektionen im Rahmen der zahnärztlichen Versorgung jedoch nur unzureichend bestimmt.

**Methodik:**

Ein systematisches Review und Metaanalyse wurde von allen Studien durchgeführt, die vor Mai 2023 veröffentlicht wurden und epidemiologische Daten zum Auftreten von SARS-CoV-2 bei ZP zur Verfügung stellten. Die dokumentierten Prävalenzen wurden mithilfe eines Random-Effects-Modells gepoolt und Odds Ratios (OR) mit 95%-Konfidenzintervallen (95%-KI) berechnet. Einflussfaktoren wurden narrativ bewertet. Das Risiko von Verzerrungen wurde mithilfe des Tools für Prävalenzstudien des Joanna Briggs Institute evaluiert.

**Ergebnisse:**

Neunundzwanzig Studien mit insgesamt 85.274 zahnmedizinisch tätigen Personen erfüllten die Einschlusskriterien; hiervon wurden 27 als Prävalenzstudien einer Metaanalyse unterzogen. Die Gesamtprävalenz von SARS-CoV-2 bei ZP betrug insgesamt 11,8% (13.155/85.274; 95%-KI 7,5%–17 %), wobei der Grad der Heterogenität zwischen den Studien beträchtlich war (I^2^=99,7%). Die gepoolte Prävalenz betrug bei Zahnärzten und Dentalhygienikern 12,7 % (1943/20.860; 95%-KI 8,0%–18,0%), was im Vergleich zur Prävalenz bei zahnärztlichem Hilfspersonal ein deutlich erhöhtes SARS-CoV-2-Infektionsrisiko aufzeigt. Dort betrug die gepoolte Prävalenz mit 5,2% weniger als die Hälfte (613/15.066; OR=2,42; 95 %-KI 2,2–2,7).

In der Untergruppe von 17 Studien aus Ländern mit hohem Einkommen war die Prävalenz mit 7,3% (95%-KI, 5%–10 %) bei ZP erheblich niedriger als in Ländern mit niedrigem und mittlerem Einkommen mit 20,8% (95%-KI: 14%–29%; p<0,001). In 19 der 29 Studien (65,5%) fehlten konkrete Angaben zur Verwendung bzw. zur Adhärenz persönlicher Schutzmaßnahmen, während in Publikationen mit konkreten Zahlangaben das Tragen von N95- (oder zumindest OP-) Masken mit einem geringeren SARS-CoV-2-Infektionsrisiko bei ZP assoziiert erscheint.

**Schlussfolgerungen:**

Zahnmedizinisches Personal war, in Abhängigkeit von seiner Nähe zu Patienten, während der COVID-19-Pandemie einem besonderen SARS-CoV-2-Infektionsrisiko ausgesetzt. Bis ein signifikanter Impfschutz gegen neuere SARS-CoV-2-Varianten in der Bevölkerung aufgebaut werden kann, sollten zahnmedizinische Einrichtungen weiterhin ihren Fokus auf den Einsatz von persönlichen Schutzmaßnahmen gemäß den aktuellen Richtlinien legen.

## Introduction

Since its emergence in 2020, the Coronavirus disease-2019 (COVID-19), with its underlying pathogen acute respiratory syndrome coronavirus type 2 (SARS-CoV-2), has caused nearly 7 million deaths worldwide [1]. It clearly outranked tuberculosis (TB) during its pandemic run as the deadliest infectious disease [2]. By comparison, TB took the lives of 1.6 million, with a slight uptick in its decades-long trend. Aggravating its impact, SARS-CoV-2 infection appears to lead to postacute sequelae in up to 23% of all individuals [3]. SARS-CoV-2 is spread either person-to-person or indirectly via respiratory droplets and aerosols. Multiple publications in the scientific body discuss the risk of dental health care workers of contracting COVID-19 following procedures such as osteotomies, drilling, prophylaxis, and ultrasonic scaling where, due to the nature of the activity, direct contact with the aerosols produced cannot be avoided by spatial distancing [4], [5], [6]. Therefore, the daily use of personal protective equipment (PPE) in each procedure, consisting in the use of disposable gloves, protective glasses, and especially face masks, is critical to the avoidance of aspiration of virus particles.

The reiterated recommendation that PPE be used systematically in professional patient-related activities must be considered in light of existing guiding principles that did not start with COVID-19. The first H1N1 influenza pandemic, which emerged in 2009, led to CDC recommendations for respirator use as preventive measure against virus transmission when caring for any patient presumedly infected [7]. Since then, in the years immediately following, several high-quality studies have proven that adequate PPE reduces the risk of pandemic influenza A (H1N1) infection in healthcare workers [8], [9], [10]. That said, the 2019 ResPECT study, with its pragmatic, cluster-randomized approach, failed to document any superiority for this purpose of N95 respirators to simple medical masks [11].

Organizational efforts to implement such protective measures in dental practices have been highly encouraged, and advanced methods of empowering and motivating DCW to respect the requirement for PPE under challenging circumstances have been widely discussed [12], [13]. Still, the association between the systematic use of PPE in DCW and the risk of becoming SARS-CoV-2 infected, delineating DCW subgroups and other, non-dental HCW, have to date been insufficiently investigated. Moreover, global prevalence in DCW has been systematically examined only in Bitencourt’s 2022 review [14] which itself included only studies published prior to April 2022 and did not include an in-depth analysis of the use of preventive measures. For this reason, we conducted an update of reports on the risk of SARS-CoV-2 transmission to caregivers in dental healthcare settings, now with a particular emphasis on infection control practices, in the examined facilities.

## Methods

### Search strategy

We performed a systematic search of electronic databases (PubMed, Embase, Web of Science, Cochrane). Search strategies combined relevant terms for SARS-CoV-2 infections in DCW with those for the occurrence, i.e., prevalence and incidence (Table 1 (Tab. 1)). The search called for all records published in the English language, up to April 24, 2023, with no geographic restriction applied.

### Study selection 

Original articles in peer-reviewed journals reporting the prevalence and/or incidence of SARS-CoV-2 infections (or sufficient data to calculate them) in DCW were eligible for inclusion, whereas review articles and conference abstracts were excluded. If there were studies reporting duplicate data, the study with the most up-to-date and complete data was used. Reference lists of the included articles as well as of the review articles were manually screened to check for additional relevant articles. All records were transferred into the EndNote reference manager, which automatically removed duplicates. The Preferred Reporting Items for Systematic Reviews and Meta-Analysis (PRISMA) standards 2021 guidelines were followed [15]. 

### Data extraction

Relevant data were extracted using a standardized data collection form and included information on location, study design, study population, sampling of study participants, data collection period, age and gender, comparison groups (if any), SARS-CoV-2 prevalence and/or incidence in the primary target group, implementation of PPE, prevalences or incidence in subgroups and/or the general population, and possible biases or confounders. 

Following the review methodology, two independent raters performed all title/abstract and full-text screening and were responsible for retrieving, extracting and checking for data eligibility. Any discrepancies were resolved by a third, independent researcher, by joint discussion.

### Statistical analysis and data synthesis

Statistical analysis was performed with metric variables expressed as means or medians, the interquartile range (IQR), i.e., the region between the 75^th^ and 25^th^ percentile, and categorical variables as absolute numbers and percentage of data entries. Univariate analyses were performed using chi-square test for categorical variables. Odds ratios (OR) as effect estimates and 95% confidence intervals (95% CI) were calculated in subgroup analysis, if appropriate. 

To arrive at pooled prevalence ratios, meta-analysis was performed based on all eligible studies for which a prevalence could be calculated: combined (all DCW), by subgroups (dentists or dental hygienists versus other DCW) and by low- or middle- and high-income countries as classified by the World Bank on July 1, 2022 [16]. As dental hygienists typically perform dental cleanings and provide preventive dental care they are not classified as dental assistants or auxiliary personnel. Dental assistants, on the other hand, have a different role. They primarily provide support to dentists in various ways, such as preparing patients for treatment, sterilizing instruments, and assisting during dental procedures. In the meta-analysis of the two subgroups of dentists versus ancillary staff, the two studies dealing exclusively with SARS-CoV-2 infection in dental hygienists were assigned to dentists, and not to ancillary staff. 

The pooled prevalence was estimated using a random-effects model considering that the prevalence of most medical conditions varies geographically and over time. An I² test was performed to quantify the heterogeneity between studies. A funnel plot of the estimated prevalence versus the margin of error (half-length of the 95% CI) was built to graphically demonstrate the variability of the study-specific estimates as a function of their estimated precision. All metanalyses were performed in StatsDirect Software, Version 3.3.6 (23.5.2023).

### Assessment of study quality

Study quality was assessed using the Joanna Briggs Institute (JBI) Critical Appraisal of Prevalence Studies scale [17], [18]. The checklist consists of nine items:

1. adequacy of the sampling frame,
2. appropriateness of the sampling method,
3. adequacy of the sample size,
4. proper description of the study participants and the setting,
5. sufficient coverage of the identified sample,
6. use of valid methods to identify the infection,
7. standard and reliable method for measuring the infection in all participants,
8. appropriate statistical analysis, and
9. adequate response rate [19].

Each study was assessed on each of these topics and the results reported as “yes” (1), “no” (0), or “unclear” (U). An overall score was assigned to the studies, adding up the number of questions answered as Yes (maximum 9). Studies were categorized as having a “low risk of bias”, i.e., high-quality study, if they accumulated at least seven items answered as “yes”. Studies were classified as having a “moderate risk of bias” when a study was scored with only four to six “yes” answers [19].

## Results

**Figure 1:**
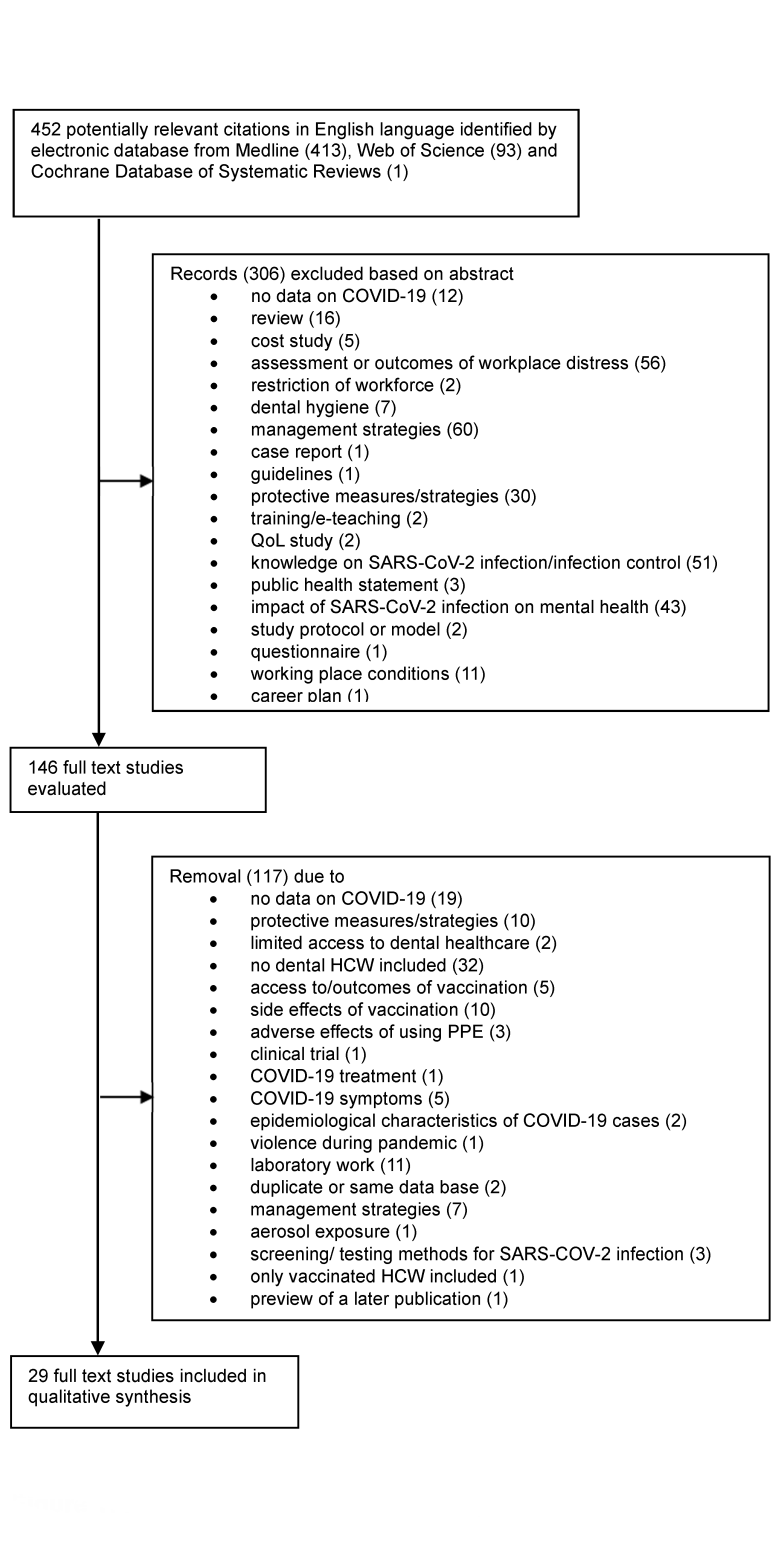
PRISMA flow diagram of study selection

Figure 1 (Fig. 1) presents a flow-diagram of the literature search results. A total of 452 articles in English language were obtained through database searching. Strictly following our inclusion criteria as described above, 29 studies were eligible for inclusion [20], [21], [22], [23], [24], [25], [26], [27], [28], [29], [30], [31], [32], [33], [34], [35], [36], [37], [38], [39], [40], [41], [42], [43], [44], [45], [46], [47], [48]. The characteristics of the included studies, are presented in Attachment 1 (Study design and sociodemographic characteristics) and Attachment 2 (Risk assessment). 

### Study characteristics

Twenty two out of the total of 29 studies had a cross-sectional design (76%), while seven studies were longitudinal cohort studies (24%). Overall, three registry-based studies were included [20], [24], [39]. The sample size ranged from 20 to 48.301 DCW. Prevalence rates for SARS-CoV-2 infection among participants varied from 0.25% (Bonta et al. [26]) to 43.9% (Suarez-Cabello et al. [48]). Fifteen studies (52%) mentioned reference data from the general population in their study. 

The included studies came from a total of 20 countries. The largest number came from Brazil with 4/29 (13.8%) and the USA 3/29 (10.3%). Italy, Poland, Canada and the UK each accounted for 2/29 (6.9%). The other countries with a single publication were Germany, Russia, Qatar, Saudi- Arabia, Spain, Sweden, Iraq, France, Romania, Argentina, Peru, Czech Republic, Iran and Norway, equivalent to 3.4% each. More than half of those (13/29, or 44.8%) were conducted in high-income, all other studies were conducted in low- or middle-income countries.

**Figure 2:**
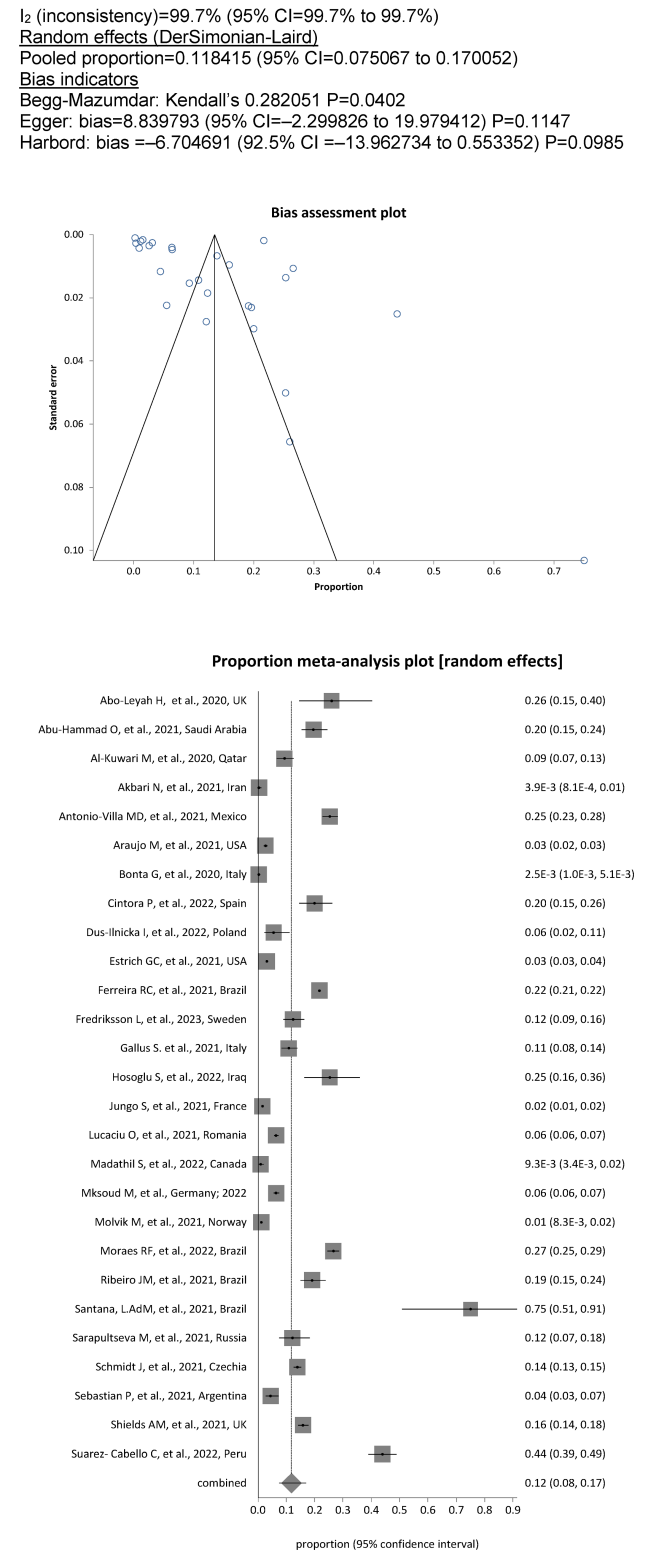
Figure 2. Pooled SARS-COV-2 prevalence in all DCW

**Figure 3:**
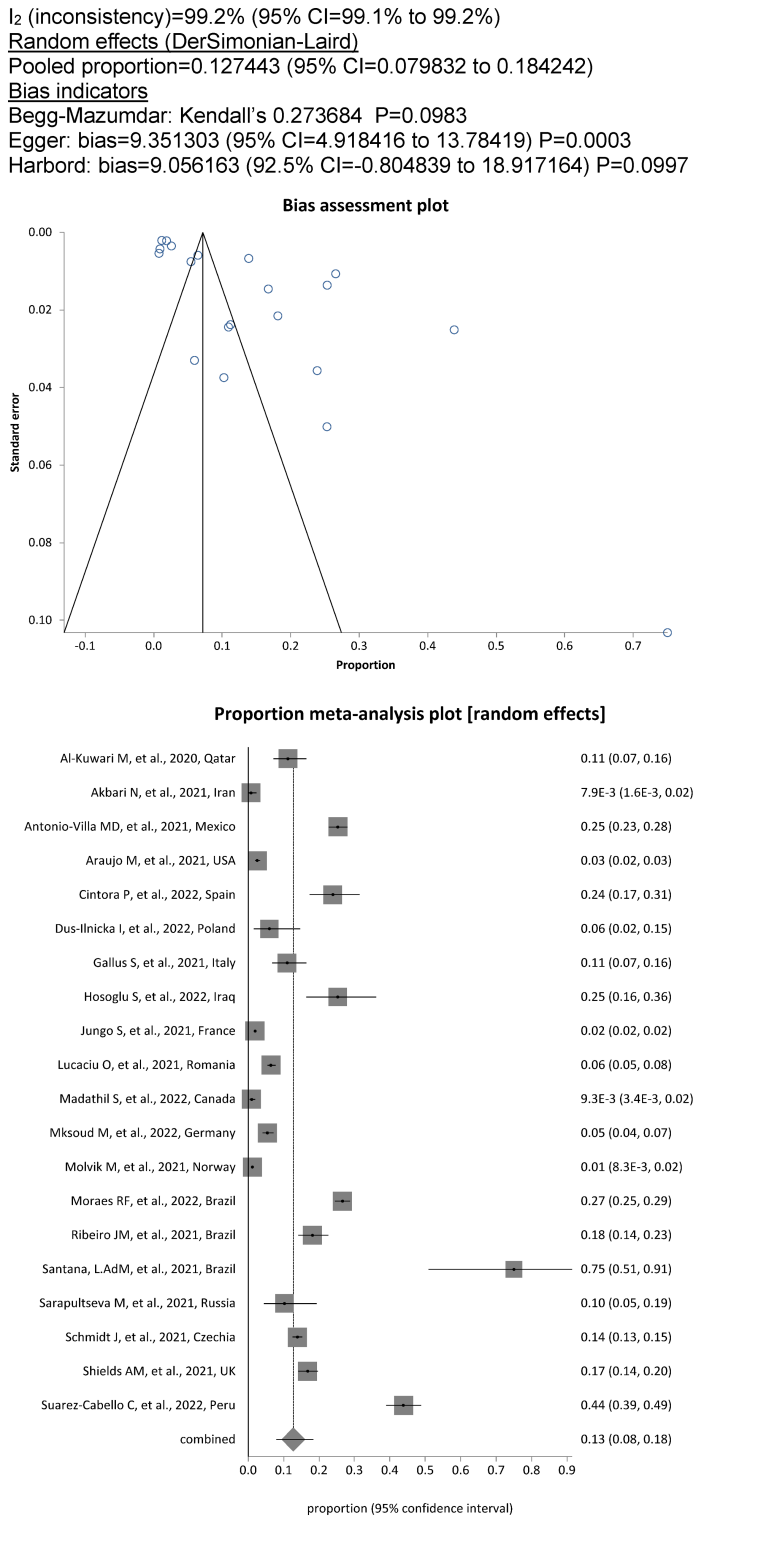
Figure 3. Pooled SARS-COV-2 prevalence in dentists and dental hygienists

**Figure 4:**
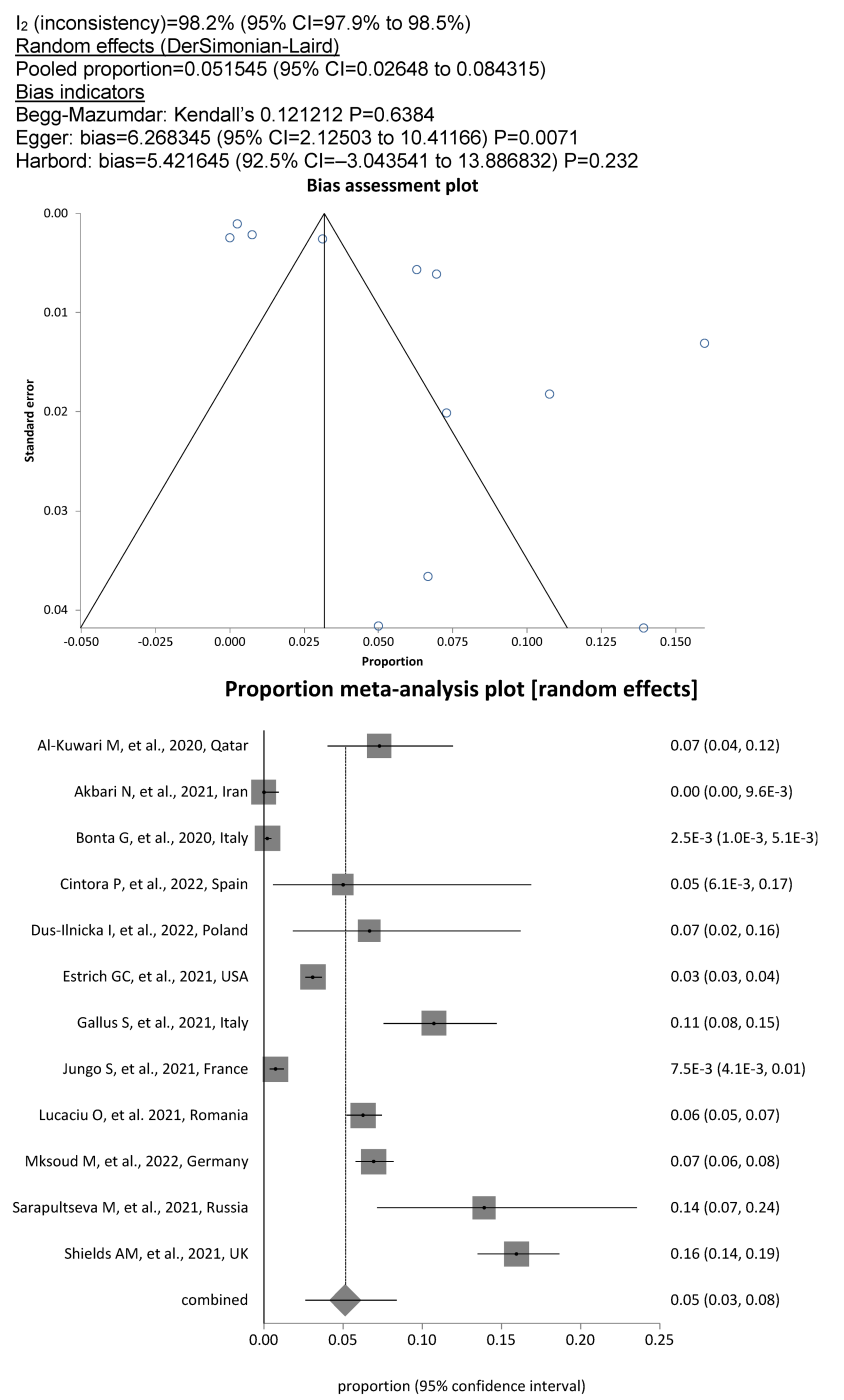
Figure 4. Pooled SARS-COV-2 prevalence in dental assistants

Most studies, 19/29, or 65.5%, investigated SARS-COV-2 infections in DCW in the pre-vaccination period in 2020 only, three studies included the first months of 2021 in their observation period, five studies, or 17.2%, came from 2021, two studies started in 2022 and one started in December 2020 with follow-up questionnaires to be answered in January 2022. With exception of Rock et al. [42], who only calculated the cumulative incidence rate, and of Froum et al. [32], who provided no figure of exposed employees as denumerator, for the remaining 27 studies the proportion of SARS-CoV-2 infections in the study population of interest at a specific point or a specific period could be calculated. Analysis was based either exclusively on web-based questionnaires (11 studies), regular surveillance databases (4 studies), evaluating RT-PCR testing within the framework of self-selected observation studies (five studies) or determining the presence of IgG/IgM antibodies against spike proteins of SARS-CoV-2 (8 studies) by ELISA-testing (see Attachment 2).

met the criteria for the meta-analysis. Among the included DCW, the overall prevalence of SARS-CoV-2 was 11.8% (13,155/85,274; 95%CI, 7.5%–17%), whereby the degree of heterogeneity between the studies was considerable (I^2^=99.7%; Figure 2 (Fig. 2)). The pooled prevalence rate for dentists and dental hygienists alone was 12.7% (1943/20,860; 95% CI, 8.0%–18.0%; Figure 3 (Fig. 3)), showing significantly increased odds of contracting a SARS-CoV-2 infection for this personnel compared to assistant personnel, the prevalence rate for which was less than half, at 5.2% (613/15,066; OR=2.42; 95% CI, 2.2–2.7; Figure 4 (Fig. 4)). 

**Figure 5:**
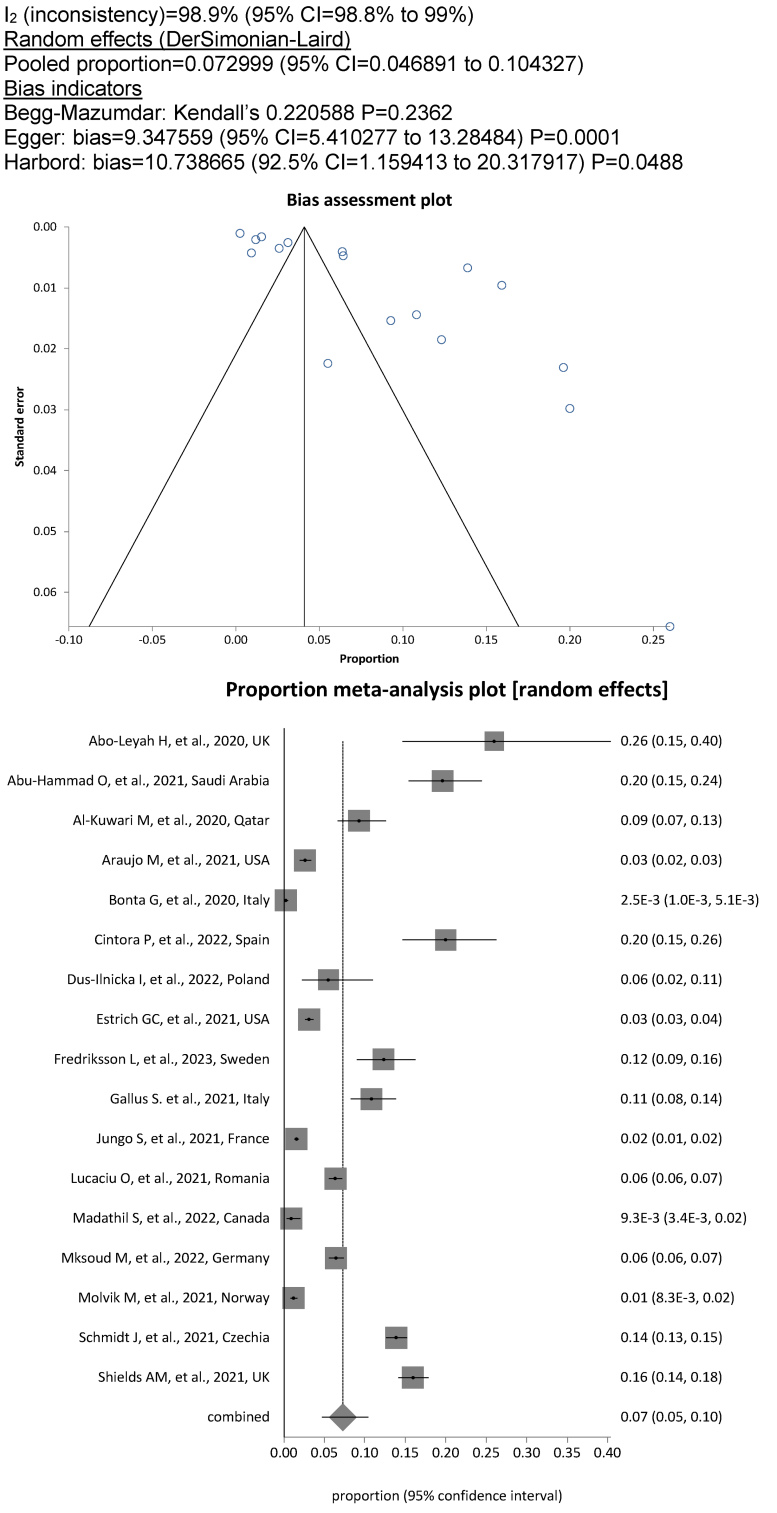
Pooled SARS-COV-2 prevalence in DCW from high-income countries

**Figure 6:**
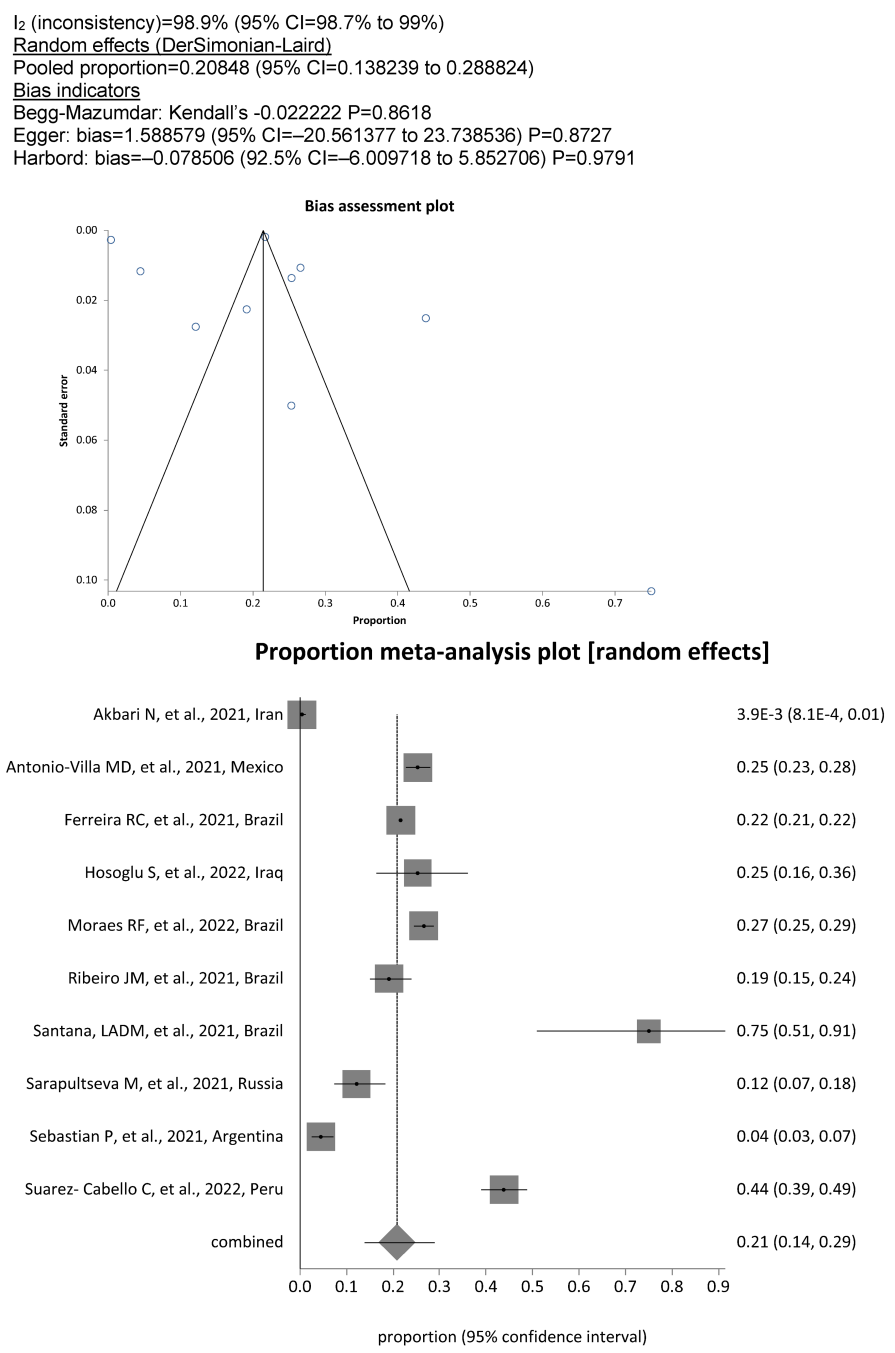
Pooled SARS-COV-2 prevalence in DCW from low- and middle-income countries

Utilizing the World Bank’s assessment of national income levels, a sub-group analysis was performed. In the subgroup of 17 studies coming from countries with high income according to the World Bank criteria there was a low pooled prevalence of 7.3% (95% CI, 5%–10%; Figure 5 (Fig. 5)). In contrast, pooled prevalence from the remaining 10 studies performed in low- or middle-income countries increased to 20.8% (95% CI, 14%–29%; Figure 6 (Fig. 6)). The difference in SARS-CoV-2 prevalence as shown in high- and low- or middle-income countries was highly significant (5.1% [1634/32,053] vs 21.6% [11,521/53,221], P<.0001).

### Comparison of SARS-CoV-2 prevalences in DCW to the general population

No significant difference between the prevalence rates found for dentists and those found for the general population was the conclusion in four studies [31], [35],[38], [41]. In seven studies, however, the SARS-CoV-2 prevalence was significantly higher for dentists. Only in Schmidt’s study [45] was the prevalence lower for dentists vs the general population during the observation period, but information on the true prevalence was not available because 154 of the 2,716 participants, or 5.7%, were not tested by PCR. Therefore, despite their having typical clinical symptoms, the authors could not consider them in their evaluation. In the three studies comparing incidence rates, the incidence among dentists was both higher [30] and lower [37], [42] as compared to the general population. In Rock’s longitudinal study [42] following a cohort of SARS-CoV-2-naïve Canadian dentists from December 2020 through January 2022, the incidence was less than half that of the population in the selected provinces (2.39% versus 5.12%). Here, the matter of vaccination coverage must be considered. More than two thirds (69%) of the dentists queried had received 2 vaccine doses by the end of the study period, whereas coverage data for the general population being compared was not reported.

### Comparison of SARS-CoV-2 prevalences between DCW and non-dental medical personnel (HCW) 

In four of the 29 studies, the SARS-CoV-2 prevalence could be calculated for DCW versus non-dental HCW, with no significant difference found in 2 studies [20], [39]. In two studies [22], [24], the prevalence in DCW was significantly lower.

### Comparison of SARS-CoV-2 prevalences between subgroups

In 12 studies, SARS-CoV-2 rates in dentists were compared to rates in other DCW. In ten studies no statistical significant differences could be found [20], [23], [28], [31], [33], [36], [38], [44], [46], [47]. In Cintora et al. [27] a nearly five-fold higher chance to be SARS-CoV-2 infected was assessed in doctors compared to members of the administrative staff (24% vs 5%; OR 5.96 [95% CI, 1.4–25.9]). Jungo et al. [35] found a prevalence rate in dentists twice that found for dental assistants (1.9% versus 0.8%; OR 2.56 [95% CI, 1.44–4.52]). 

### Comparison of SARS-CoV-2 prevalence rates within specific groups

To answer the question of whether the level of professional experience would be associated with better precautionary measures, Bonta et al. [26] clustered dental hygienists in three years-professional-experience groups, reflecting their decades of activity in the profession. 

The COVID-19 prevalence in the cohort was so low during the study period of May 2020 (7/2798; 0.25%) that no statistical difference could be seen. In Cintora’s multivariate analysis [27] of prevalence in doctors working directly with patients, in which administrative dental staff was taken as the reference, only orthodontists among the five different subgroups of doctors had a higher (more than ten-fold higher) chance of being infected (OR 10.13 [95% CI, 2.25 to 45.68). In Estrich’s study [29], the use of PPE increased significantly with the duration of employment, divided into blocks of 10 years, from 54.6% to 60.7%. Hosoglu et al. [34] found a high SARS-CoV-2 infection rate of 25.3% in Iraqi dentists and demonstrated a nearly seven-fold higher chance of being infected (OR 6.9 [95% CI, 1.2 to 37.6] among dentists working in a public hospital with a relatively high frequency of contact to patients vs those in private practice. This was the only risk factor identified for SARS-CoV-2 infection in that study. In Ribeiro’s study [41] SARS-CoV-2 prevalence in dentists was associated with treatment of patients with fever (OR 2.99 [95% CI, 1.03–8.7]), but also with having a COVID-19 case in the one’s own household (OR 2.5 [95% CI, 1.12–5.3]). 

### Impact of the use of and adherence to personal protective equipment (PPE)

In 19 studies, beyond general references to applicable guidelines or vague hints, no specific information on the use of PPE and adherence to PPE guidelines could be found [20], [21], [22], [23], [24], [27], [30], [31], [33], [36], [39], [41], [42], [43], [44], [45], [46], [47], [48]. Furthermore, Hosoglu’s study [34] indicates how many dentists wore protective goggles and washed their hands, and Dus-Ilnicka’s study [28] describes numerous protective measures, e.g., gravitational ventilation, but in both studies, no data were found on the wearing of face masks. 

In Abo-Leyah’s study [20] on Scottish healthcare workers, the prevalence in staff members working in critical care areas was 16%, indicating insufficient measures to protect even high-risk front-line staff. Abu-Hamad et al. [21], Hosoglu et al. [34], Lucaciu et al. [36] and Santana et al. [43] themselves, all state that the insufficient use of PPE may be responsible for the high number of SARS-CoV-2 infections. Ribeiro et al. [41] mention a rigorous use of PPE in dentists only when treating patients with fever, and Ferreira et al. [30] explicitly underline the need of establishing PPE given the 5% higher incidence of SARS-CoV-2 infections among dental DCW vs other HCW. 

In contrast, Al Kuwari et al. [22] and Akbari et al. [23] state that the low SARS-CoV-2 prevalence in DCW compared to the general population may be attributed to the proper use of PPE. In Shields’s longitudinal study [47], baseline seroprevalence in 1507 DCW of the West Midlands who had been recruited in June 2020 was 16.3% compared to estimates in the regional population of 6% to 7%. In the follow-up between June 2020 and January 2021, however, seroprevalence in those DCW who had been seronegative at baseline fell to 11.7% while background population levels remained stable. According to the authors, this reduction was associated with enhanced use of PPE, including FFP3 masks, over those six months.

In the very few studies with concrete figures on implementation of PPE, the wearing of N95 (or at least surgical masks) appears to be associated with low SARS-CoV-2 prevalence rates. Maximal protection was provided by the various protective measures introduced in three dental offices in New York [32]. There, despite exposure towards 2,820 patients in the six months between March and September 2020, no member of the staff became SARS-CoV-2 infected. To achieve this, not only wearing of FFP2 masks, but also mandatory hand washing, HPC air filters and UV-C germicidal lights were required. They reported that the risk of transmission by any patient may also be reduced by a preselection of patients who had to answer whether they had had contact with COVID-19 persons, were previously tested positive or currently had fever. A limitation to be considered is that the number of exposed staff members was not revealed. In US study of Araujo et al. [25], where a SARS-CoV-2 prevalence as low as 2.6% was assessed in U.S. dentists, dentists showed a high level of adherence to recommendations despite of a shift from 99.4% in the first survey to 88% adherence in the final one. 

A particularly low prevalence was found in the survey of dentists in Lombard, the region with the highest number of SARS-CoV-2 infections in Italy at that time [49] conducted in May 2020 [26]. In that study, reporting 0.25% SARS-CoV-2 infections, 82.5% of dental hygienists had worn surgical masks and 90.6% protective glasses or visors. In Madathil’s Canadian study [37], which covered a study period from July 29, 2020, through February 12, 2021, nearly all dentists were using either N95 respirators or surgical masks. Of note, the incidence proportion was lower with 1,084 per 100,000 of dentists as that of the general population at 1,864 per 100,000 people). 

Mksoud’s seroepidemiological study [38], which included IgG antibody sampling more than one year after the start of the COVID-19 pandemic in Germany, showed that while only three quarters (74.2%) of DCW wore FFP masks, no difference in their SARS-CoV-2 prevalence could be shown to that of the general German population. Moraes et al. [40], who found a prevalence of 27% among Brazil dentists, reported that only 69% of dentists wore N95 masks as of May 2021. 

In Estrich’s study [29], in which the SARS-CoV-2 prevalence of 3.1% in dental hygienists was higher than that of the US population at large (2.3%) over the same period, only slightly more than half (55.7%) of all dentist hygienists consistently used PPE as required by the CDC. Surprisingly, the PPE usage rate among hygienists with professional experience exceeding 21 years came in at only 60.7% 

In Juno’s study [35] covering 6,040 French dentists or assistants, only symptomatic dentists and assistants were evaluated. In this subgroup, significantly fewer assistants wore FFP2 masks than dentists (3.9% vs 8.8%; p<0.01) and safety goggles (39.2% vs 62.0%; p<0.001). 

### Study quality

The studies were assessed to have generally good quality, with a mean average critical appraisal score across all studies of 8 out of 9 (Attachment 3). The question that affected the scores the most was ‘Was the sample frame appropriate to address the target population’ as in 10 studies selection bias was possible. Furthermore, in five studies where no sample size calculation had been performed at the outset, the response rate was low raising concern about the representativeness of those studies. That offers the possibility that the number of SARS-CoV-2 infections in the respondents differed significantly from those of the non-respondents. However, there is no apparent reason to suppose that the losses had any systematic direction. In Schmidt’s study [45], an underreporting bias cannot not be excluded as not all participants showing symptoms indicative of COVID-19 were tested. 

## Discussion

This analysis investigates the meaningfulness of the burden of COVID-19 in dental health workers with respect to epidemiological indicators. 

Since most of the publications available today cover the period of the COVID-19 pandemic in which vaccination was not yet available, the influence of the use or lack of personal protective measures in dental practice becomes particularly clear. Considering that professional protective measures among medical personnel had already been recommended in the context of previous viral outbreaks, such as the SARS epidemic of 2003 or the 2009 H1N1 influenza pandemic [7], [50], a comparable or lower prevalence in dental facilities than in the respective general populations of the countries of origin of the studies would have been expected, especially in the initial phase of the COVID-19 pandemic. Those studies that were conducted in the first months of the pandemic demonstrate, unless figures were biased by factors such as lack of testing or early vaccination in health care settings, a comparable or higher prevalence of SARS-CoV-2 infection in DCW than in the general population, suggesting that the use of PPE, especially the wearing of face masks, was at the time not sufficiently implemented to mitigate the suddenly increased infection risk that DCW were facing. This is particularly true for facilities in low- or middle-income countries, where poor or delayed availability of PPE, especially protective masks, could be assumed for reasons of acquisition cost or logistics. Regardless of external comparisons or cost structure, however, the significantly higher prevalence among dentists and dental hygienists compared to assistants clearly shows the particularly high risk of SARS-CoV-2 infections for DCW with close patient contact.

The selection process within the framework of this systematic review showed that studies focusing on the prevalence and incidence of COVID-19 in DCW, and especially information on possible underlying factors, are scarce. Most studies focus on the perception of COVID-19, associated mental stress and disabilities due to COVID-19. Others describe the type of protective and hygienic measures employed. Among thousands of COVID-19 studies, only 29 could be identified that focused on the prevalence and incidence of SARS-CoV-2 infections in DCW. The results of our study, as far as the COVID-19 pre-vaccination area is concerned, confirm first of all the generally increased occupational risk of infection that DCW face compared to the risk of the general population worldwide. This applies equally to low-income and high-income countries, although the pooled prevalence in low-and middle-income countries is almost three times higher at 20.8% vs 7.3% for DCW in high-income countries, with the more limited availability of costly PPE presumably contributing to the effect. As the meta-analysis of studies with prevalence data shows, dentists or dental hygienists are significantly more affected by SARS-CoV-2 infections than assistant or administrative staff. While the latter group had a pooled prevalence of only 5.2%, the prevalence among dentists and dental hygienists was more than twice as high at 12.7%. The much higher prevalence in dentists suggests a direct increase of infection risk with increasing proximity to aerosols from COVID-19 patients. This is true for all exposed individuals, and so the difference in outcome rates can be explained by the different nature of patient interaction in a practice. The dentist or dental hygienist is in close proximity to the patient's face, which increases the risk of virus transmission, as he/she is exposed to the patient’s respiratory secretions for almost the entire contact period. The dental assistants, on the other hand, are only temporarily exposed, not for the entire duration of the treatment. Their close contact with the patient is limited, as the scope of their duties also extends to the documentation of treatment as well as the preparation and post-processing of instruments. Accordingly, they primarily carry out supporting activities in relation to the treatment process and are not as exposed as the dentist self. Nevertheless, it is crucial that both the dentist and the dental assistant continue to follow strict protective measures, such as utilizing PPE and following strict hygiene protocols, to hold the risk of infection to a minimum. 

With regard to this issue, although the studies cannot be directly compared to each other, our analysis shows a nearly linear impact between the use of PPE and adherence to PPE guidelines and SARS-CoV-2 prevalences. This especially concerns the wearing of any face masks, not necessarily FFP2 masks. Furthermore, the importance of using PPE by DCW is not only evident in the context of the SARS-CoV-2 infection risk. According to the results of the Northern German StaphDent study the consistent use of PPE has also been proven to be protective in the colonization of MRSA [51]. Another easily implemented measure to prevent the transmission of SARS-CoV-2 to DCW, namely the preventive use of virucidal gargling solutions by patients before their dental treatment [52], [53], is also not been mentioned in the studies included here. Of note, since March 2020, pre-exposure prophylaxis has been carried out at the German Greifswald University Medicine with 1.25% aqueous PVP iodine solution and, in case of contraindication, with the combination ethanol/essential oils. Since then, there has not been one case of intolerance and no SARS-CoV-2 transmission from patients to the physician or dentist [54]. 

Mksoud’s German seroepidemiological study [38] showed one quarter of DCW ignoring the need to wear FFP masks, even one year into the pandemic. This shows that the DCW problem by no means is limited to DCW in low-income countries, and that it requires continuous efforts to persuade those responsible to implement and/or enforce the existing guidelines and recommendations in full. 

At first glance, this task no longer seems urgent. This is due to the fact that following a recommendation of its COVID-19 emergency committee, the World Health Organization (WHO) on 5 May 2023 announced that COVID-19 is no longer a public health emergency of international concern [55]. This, however, does not mean that preventing the spread of SARS-CoV-2 should be dropped as a public health priority. Although we – in a snapshot of the moment – are not facing new variants of concern [56], the WHO has emphasized the importance of continuing to limit SARS-CoV-2 transmission and of treating patients with COVID-19 to reduce mortality and morbidity [57]. At the time of writing (16 October 2023), a total of 13,516,459,649 vaccine doses have been administered worldwide. Still, the WHO reports a number of 31,939 new official weekly diagnoses [58] that may be largely underestimated due to a lack of regular testing and/or reporting [59]. In Germany, for example, on February 28, 2023, COVID-19 testing was dropped from coverage by public funding [60] and the Robert Koch Institute’s COVID-19 Dashboard, which provided an overview of new corona infections, deaths, and 7-day incidence, was discontinued on June 6, 2023. 

As SARS-CoV-2 XBB strains (a subgroup of Omicron) currently predominate globally, including in the EU/EEA countries, EMA and ECDC have most recently recommended XBB.1.5-adapted COVID-19 vaccines [61]. These new monovalent vaccines can be used for both basic immunization and booster shots. The effectiveness of the new vaccination recommendations for protection against SARS-CoV-2, however, will remain in the foreseeable future the subject of continual scientific evaluations. As admissions to hospitals have shown steadily increasing trends over recent weeks in eight EU countries [62], a significant level of vaccination protection against omicron and newer SARS-CoV-2 variants must be built up in the population again. Until then, the most important mitigation measure for occupational risk remains the protection by other hygiene measures, especially through masks, particularly during the colder months of the year. 

This analysis has several limitations. First, eight of the studies were single center studies and, although a considerable number of participants was observed, it remains uncertain whether the results can be projected onto the collective DCW in the respective countries. 

Second, self-reporting of SARS-CoV-2 testing was considered in calculating prevalence and crosschecking by health care professionals was not possible in most cases, so that occasional misstatements cannot be ruled out. On the other hand, the observed selection bias in ten studies and the response bias in five studies suggests that the reported infection numbers are under- rather than overestimated. 

Third, in many cases, any indication of the availability and use of PPE over the study period is lacking, so any relationship between prevalence and the use of PPE use could either only be suggested by indirect information from the text or was limited to the personal assessment of the authors. 

Fourth, as the high I^2^ value in our meta-analysis suggests significant heterogeneity, and not all of the studies differentiated between dentists or dental hygienists and other DCW, the pooled prevalence values calculated for these subgroups must be interpreted with some caution. However, the higher odds for dentists and/or dental hygienists of contracting a SARS-CoV-2 infection as compared to those of assisting personnel appear to be convincing, despite the multiple differences between the single studies. We note here emphatically the differences in study period, study design, and the settings, due to 20 different countries, in which the studies were conducted. 

In conclusion, it is important that the vigilance of dental personnel towards infection prevention is maintained and that hygiene measures are not considered an outdated practice. Discontinuation of PPE could easily pave the way for spreading of the virus in the DCW community, as the studies in this review have shown, and may place an even greater burden on the health care system. 

## Notes

### Competing interests

The authors declare that they have no competing interests.

### Author’s ORCID

- Roland Diel: 0000-0001-8304-7709
- Albert Nienhaus: 0000-0003-1881-7302

## Supplementary Material

Table S1: Study design and sociodemographic characteristics of study participants

Table S2: Risk assessement

Table S3: Joanna Briggs Institute (JBI) critical appraisal tool for prevalence studies

## Figures and Tables

**Table 1:**
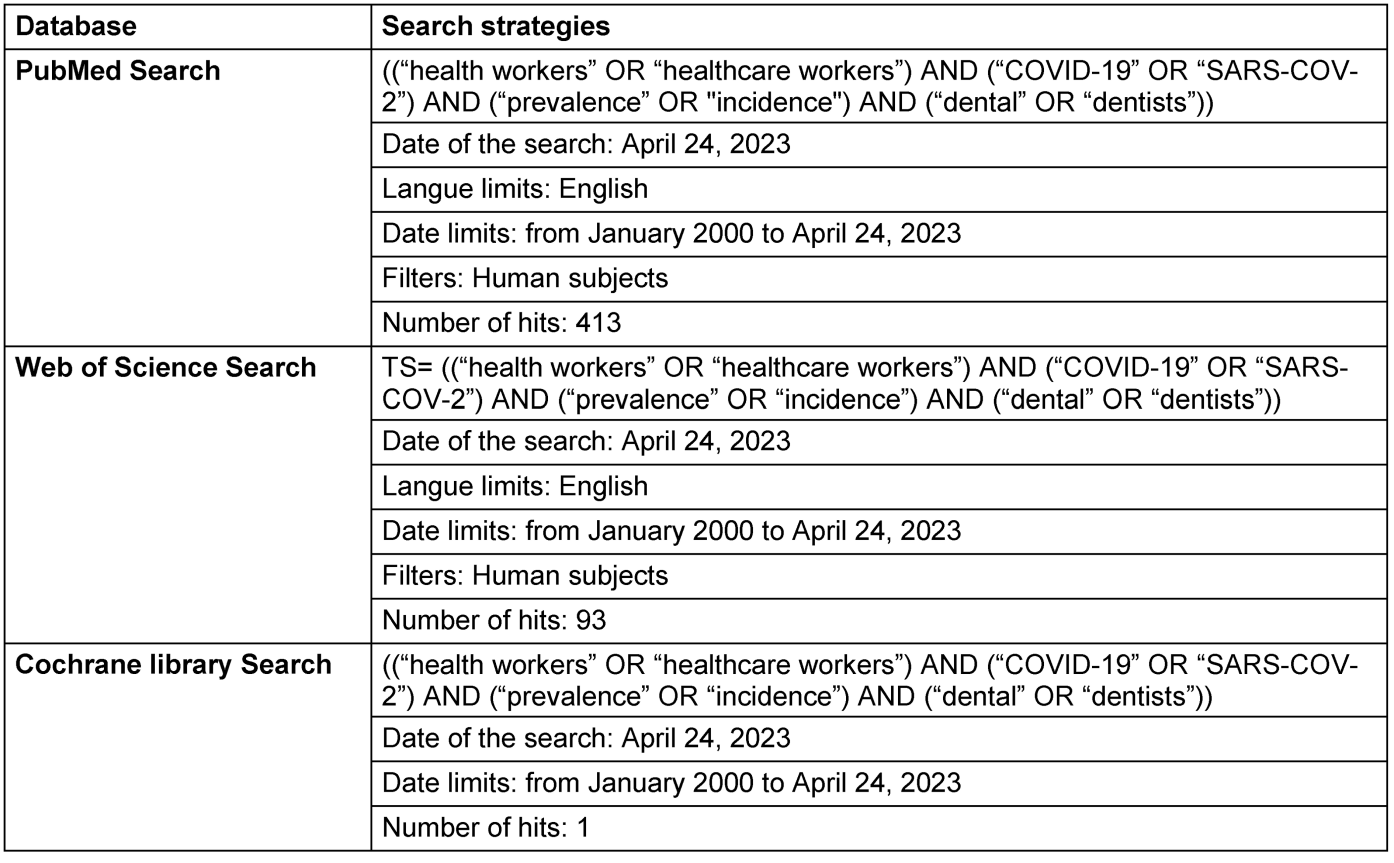
Database search strategies
