# Supplementary material for: Risk of SARS-CoV-2 infection in dental healthcare workers – a systematic review and meta-analysis
Source: GMS Hyg Infect Control. 2024 Mar 5;19:Doc09. doi: 10.3205/dgkh000464 (PMC11035909; doi:10.3205/dgkh000464)
Supplement: Table S1: Study design and sociodemographic characteristics of study participants [file HIC-19-09-s-001.pdf]

# Attachment 1

## Table S1: Study design and sociodemographic characteristics of study participants

| **Author, publication year, country** | **Setting** | **Study type** | **Number of participants** | **Observation period** | **Age, gender** |
|---|---|---|---|---|---|
| Abo-Leyah H, et al., 2020, UK [20] | SARS-CoV-2 infections in Scottish HCW and social care workers | Prospective longitudinal seroprevalence study (SARS-CoV-2 antibody testing) | Age >16 yr; 2063 out of 14,000 staff members invited by formal published solicitation; only 50 DCW as subgroup included | 28 May and 2 September 2020 (testing times) | All 2063 participants: Median 46 yr (no IQR provided); female 81.7%; 18.3% male; no data provided for the 50 DCW included |
| Abu-Hammad O, et al., 2021, Saudi Arabia [21] | Faculty and clinical students (4th, 5th, 6th year) at an academic dental hospital | Online cross-sectional survey; single-center study | 316/344 (91.9%) of eligible faculty and students included | Collection of data during March to August 2021 | Mean 28.88 ± 8.77 yr (range 20-55 yr);<br>female 173 (54,7%); male 143 (45,3%); |
| Al Kuwari M, et al., 2020, Qatar [22] | Primary Healthcare workers in the State of Qatar. | Cross-sectional | 7407 tested HCW (81.15% of 9127 HCW employed) | March 1st and October 31st, 2020 | Median age of the 1199 infected HCWs was 36 yr (no ICR provided); 488 (40.7%) female; 711 (59.3%) male |
| Akbari N, et al., 2021, Iran [23] | All Iranian dentists | Cross sectional; online survey (questionnaire registered at a website) | Total no. of Iranian dentists not mentioned. 400 questionnaires received by volunteers, of those 381 questionnaires were completed | 9 to 23 May 2020 | Mean 44.56 ± 11.72 yr (dentists); 34.65 ± 10,27 yr (dentists' assistants);<br>female 53.5%, male 46.5% (dentists);<br>female 96%, male 4%, (dentists' assistants) |
| Antonio-Villa MD, et al., 2021, Mexico [24] | HCW living in Mexico City | Cross-sectional Analysis (National Epidemiological Surveillance System database in Mexico City) | As of September 2020 20th, 2020, 57,758 HCWs were tested for SARS-CoV-2 and 17521 were confirmed (30.33%). | September 20th 2020 | No data for dental HCW provided. |
| Araujo M, et al., 2021, USA [25] | Licensed dentists in the United States | Prospective longitudinal cohort study (6 monthly follow-up questionnaires) | Number of invited dentists not mentioned. Of initially 2,196 participating dentists 1,802 (82.1%) were general dentists. A total of 785 (35.7%) of initial respondents finished all 6 surveys. | June 8 to November 13, 2020 | Median 52.6 yr (no IQR provided); range 27- 84 yr; female 845 (38.5%), male 1300 (59.2%) |

| Author, publication year, country | Setting | Study type | Number of participants | Observation period | Age, gender |
|---|---|---|---|---|---|
| Bonta G, et al., 2020, Italy [26] | Dental hygientis in Lombardy | Cross-sectional survey | Of 6948 questionnaires sent, 2798 dentists participated (40.27%) | Survey period from 12 May 2020 to 23 May 2020 | Age and gender not provided for the finally participating dentists |
| Cintora P, et al., 2022, Spain [27] | DCW at an academic center in Madrid | Cross-sectional seroprevalence study; blood collection on 2 dates; single center study | 155 dentists on 2 and 5 June 2020 and administrative and 40 administrative staff workers on May 11 | June and May 2020 | Mean 32.15 ± 8.97 yr (range 24-70) female 120 (61.5%), male 75 (38.5%) |
| Dus-Ilnicka I, et al., 2022, Poland [28] | Employees of the Academic Dental Polyclinic attached to the Wroclaw Medical University | Cross-sectional seroprevalence study; IgG antibody-testing | 127 healthy volunteers (3 subgroups (SUB). SUB1: dentists (n=67); SUB2: dental assistants, dental hygienists, nurses, laboratory workers (n=40); SUB3: administrative workers (n=20) | 15 March 2020 to 28 September 2020 | Mean age: SUB1: 33 ± 11.5 yr, SUB2: 48.5 ± 11.7 yr, SUB3: 44.5 ± 15.1yr; males in SUB1: 34% (n=17/50),in SUB2: 11.1% (n=4/36) , in SUB3: 42.9% (n=3/17) |
| Estrich GC, et al., 2021, USA [29] | Dental hygienists licensed in the USA (all 50 states and Puerto Rico) | Cross-sectional 30-question web-based survey | 3.6% (n=4776/ 133,000) invited dentists participated | September 29 to October 8, 2020 | Mean 44.1 ± 12.0 yr (range 18-77 yr); female 84.61% (n= 4043/4776), male 0.9% (n= 42/4776), not specified 0.8% (n=40/4776) |
| Ferreira RC, et al., 2021, Brazil [30] | e-SUS VE registry data | Cross-sectional study | 48,301 dental HCW (31,666 dentists, 16,635 technicians/ assistants | 1 January to 10 October 2020 (41 weeks) | Only age groups provided; female 10%, male 90% (dentists); female 24%, male 76% (assistant/technicians) |
| Fredriksson L, et al., 2023, Sweden [31] | Employees of a public dental service in the country of Stockholm | Cross-sectional study | 341 persons (17% of the total number of employees) randomly selected from a list | during weeks 23 to 25 in 2020 | Mean 50.1 ± 10.3 yr (range 25-71 yr); female 88.3% (n=301/341), male 11.7% (n=40/341) |
| Froum SJ, et al., 2020, USA [32] | Staff in 3 dental offices in New York (exposed to a total of 2820 patients) | Prospective study excluding persons who had contact with COVID-19 patients, were tested positive or currently had fever | Dental offices including three dentists and 3 hygienists, number of other staff members not reported (and also not answered on inquiry by mail) | 15 March to 15 September 2020 | Not provided |

| Author, publication year, country | Setting | Study type | Number of participants | Observation period | Age, gender |
|---|---|---|---|---|---|
| Gallus S, et al., 2021, Italy [33] | Dentists, dental hygienists, and dental staff in Lombardy region. | Cross-sectional study conducted on a sample of volunteers tested for the presence of SARS-CoV-2 IgM/IgG | The first 500 volunteers were enrolled in the study. Of those, valid test results were available in 499 persons. | 8 May 2020 up to 30 September 2020 | Mean 43.9 ± 14.4 yr; 41 ± 13.0 yr among women and 48.7 ± 15.7 yr among men; female 67% (n=167/499), male 33% (n=332/499) |
| Hosoglu S, et al., 2022, Iraq [34] | Dentists in Iraqi Kurdistan Region | Cross-sectional study using a structured questionnaire, spread with a snowball method among dentists | 13.8% (n=83/600) dentists in the region (31 women and 52 men) completed the questionnaire (required number from the sample size calculation fulfilled). | April 2021 | Mean 33.8 ± 6.8 yr (range 23-59 yr); female 37.4% (n=31/83), male 62.6% (n=52/83) |
| Jungo S, et al., 2021, France [35] | French dental practitioners and dental assistants | Cross-sectional online survey, distributed using a snowball sampling method | 4172 dentists and 1868 assistants (approximately 10% of French oral health-care workers) responded | April 1 and April 29, 2020 | Dentists: mean 44 yr (range 21-86 yr); Assistants: mean 38 yr (range 31-46 yr): Dentists: female 57.1%, 42.9% male: Assistants: female 98.2%, male 1.8% |
| Lucaciu O, et al,. 2021, Romania [36] | Dental practitioners from both the private and public sectors in Romania | Cross-sectional study using a web-based survey | 507 dentists (out of 16457 registered dentists) completed the survey on behalf of 3735 dental health workers working in the assessed dental offices, of those 1811 were doctors. | Survey was carried out from 26 December 2020 to 1 March 2021. | Different figures: Doctors who completed the survey on behalf of the 3735 dental health workers: female 75.3% (n=382/507), male 24.7% (n=125/507); SARS-CoV-2 positives: female 74.4% (n=177/238), male 25.6% (n=61/238) |
| Madathil S, et al., 2022, Canada [37] | Licensed dentists across Canada | Prospective cohort study; online questionnaire, sent every 4 weeks | 644 dentists responded (number of dentists required according to sample size calculation fulfilled); every 4 weeks after baseline, participants completed an online questionnaire | July 29, 2020, to February 12, 2021; median follow-up time 188 days | Mean 47.3 yr (range 24-79 yr); female 56.4% (n=363/644), male 43.6% (n=282/644) |

| Author, publication year, country | Setting | Study type | Number of participants | Observation period | Age, gender |
|---|---|---|---|---|---|
| Mksoud M, et al., 2022, Germany [38] | Dental teams in private practices across Germany | Cross-sectional (questionnaire and IgG antibody testing) | 7300 not-representative invitations to dental practices; 2784 unvaccinated dental team members from 1390 practices finally included | January to April 2021; sampling until 21 April 2021 | Mean 44.8 ± 12.5 yr; female 84%, male 16% |
| Molvik M, et al., 2021, Norway [39] | Health Service staff in Norway | Cross-sectional registry study (Beredt C19) for the whole year 2020 | 382332 HCW included (no self-employed HCW) | 2020 (whole year) | No absolute data on age provided;<br>female 83.3%, male 16.7% |
| Moraes RF, et al., 2022, Brazil [40] | Dentists from Brazil | Cross-sectional study (web-based questionnaire) | Of 24,392 invited dentists 1907 valid responses returned (7.8%); of those 1754 dentists tested for SARS-CoV-2 (1530 dentists required in sample size calculation) | Emails sent May 13, 2021 and 11 days later | No figures on age provided; female 74.1% (n=1414/1907), male 25.9% (n=493/1907) |
| Ribeiro JM, et al.,2021, Brazil [41] | Dentists from the Federal District (DF) in the Mid-west region of Brazil | Cross-sectional seroprevalence study: participants received an on-site COVID-19 IgG/IgM rapid test. | 4.15% (n=324/7900) dentists randomly selected. Younger dentists participated more often than older dentists.<br>(314 dentists required in sample size calculation) | October to November 2020 | Mean 40.2 ± 10.8 yr (range 21-71 yr);<br>female 67.0% (n=217/324), male 33.0% (n=107/324) |
| Rock LD, et al., 2022, Canada [42] | Licensed dentists in 8 Canadian provinces | Prospective longitudinal cohort study (basic and 6 follow-up questionnaires): | 876 SARS-CoV-2 negative (self-identified) dentists included (response rate only 3.15% (n=958/30444) | December 2020 through January 9, 2022 (those who had been tested positive at baseline were excluded) | Median 42 yr (25 th percentile 33, 75$^{th}$ 52); female 97.8% (n=857/876), male 2.2% (n=19/876) |
| Santana, LADM, et al., 2021, Brazil [43] | 23 maxillo-facial surgeons in a Northeast Brazilian public center (Sergipe) | Cross-sectional (collection of laboratory data of tested surgeons) | Results of 20 out of 23 maxillofacial surgeons of the hospital included. | Beginning of the outbreak until now (April 2021?) | Mean age 46.05<br>± 5.9 yr (range 38-60 yr); male/female ratio 9:1 |
| Sarapultseva M, et al., 2021, Russia [44] | 3 dental clinics in Ekaterinburg | Retrospective longitudinal study; serological testing once a week to detect IgG and IgM | 157 HCW; of those 49.7% dentists and 51.3% dental assistants | May to August 2022 | All HCW: 43.58 ± 1.66 yr; female 75.8%, male 24.2% |

| Author, publication year, country | Setting | Study type | Number of participants | Observation period | Age, gender |
|---|---|---|---|---|---|
| Schmidt J, et al., 2021, Czechia [45] | Czech dentists (chamber members) | Self-administered, cross sectional, online survey | 2716 participants of 9922 invited dentists (response rate 27.4%). 372 dentists required in sample size calculation 372. | 23 June to 4 September 2021 | Only age groups provided; female 68.9% (n=1871/2716), male 30.8% (n=837/2716), not specified 0.2% (n=6/2716) |
| Sebastian P, et al., 2021, Argentina [46] | DCW at the Dental hospital at Buenos Aires University | Cross-sectional; ELISA or RT-PCR testing | 358 of 430 (83.3%) dental workers from clinical and non-clinical areas tested during the first 180 days of COVID-19 pandemic | March to October 2020 | mean 38 ± 11 yr (range 21-59 yr); female 64% (n=228/358), male 36% (n=130/358) |
| Shields AM, et al., 2021, UK [47] | General dental practioners (GDPs) of the west midlands | Longitudinal seroprevalence study | 1507 of 1716 invited GDPs (88.3%) participated; 944 GDPs received a repeated serological analysis | June 2020; follow-up antibody testing in 944 GDPs six months after providing the baseline antibody response | Mean age 37 yr (range 29-47 yr); female 75.4% (n=1136/1507), male 24.6% (n=371/1507) |
| Suarez- Cabello C, et al., 2022, Peru [48] | Dentists in Arequipa (Southern Peru) | Observational, descriptive, cross-sectional study (web-based questionnaire) | 408 valid surveys among 3200 dentists in Arequipa (response rate approximately 12%, 344 dentists required in sample size calculation) | 1 January to 31 March 2022 | Mean 40 ± 13.4 yr;<br>female 51.0% (n=208/408), male 49% (n=200/408) |

DCW: Dental healthcare workers

GDP: General dental practioner

yr: years

## References


11. WHO. Coronavirus disease (COVID-19). Geneva: World Health Organization; 2023 [accessed 2023 Oct 26]. Available from: https://www.who.int/emergencies/diseases/novel-coronavirus-2019

2. Global tuberculosis report 2022. Geneva: World Health organization; 2022. p. 68.

3. Thaweethai T, Jolley SE, Karlson EW, Levitan EB, Levy B, McComsey GA, McCorkell L, Nadkarni GN, Parthasarathy S, Singh U, Walker TA, Selvaggi CA, Shinnick DJ, Schulte CCM, Atchley-Challenner R, Alba GA, Alicic R, Altman N, Anglin K, Argueta U, Ashktorab H, Baslet G, Bassett IV, Bateman L, Bedi B, Bhattacharyya S, Bind MA, Blomkalns AL, Bonilla H, Bush PA, Castro M, Chan J, Charney AW, Chen P, Chibnik LB, Chu HY, Clifton RG, Costantine MM, Cribbs SK, Davila Nieves SI, Deeks SG, Duven A, Emery IF, Erdmann N, Erlandson KM, Ernst KC, Farah-Abraham R, Farner CE, Feuerriegel EM, Fleurimont J, Fonseca V, Franko N, Gainer V, Gander JC, Gardner EM, Geng LN, Gibson KS, Go M, Goldman JD, Grebe H, Greenway FL, Habli M, Hafner J, Han JE, Hanson KA, Heath J, Hernandez C, Hess R, Hodder SL, Hoffman MK, Hoover SE, Huang B, Hughes BL, Jagannathan P, John J, Jordan MR, Katz SD, Kaufman ES, Kelly JD, Kelly SW, Kemp MM, Kirwan JP, Klein JD, Knox KS, Krishnan JA, Kumar A, Laiyemo AO, Lambert AA, Lanca M, Lee-Iannotti JK, Logarbo BP, Longo MT, Luciano CA, Lutrick K, Maley JH, Marathe JG, Marconi V, Marshall GD, Martin CF, Matusov Y, Mehari A, Mendez-Figueroa H, Mermelstein R, Metz TD, Morse R, Mosier J, Mouchati C, Mullington J, Murphy SN, Neuman RB, Nikolich JZ, Ofotokun I, Ojemakinde E, Palatnik A, Palomares K, Parimon T, Parry S, Patterson JE, Patterson TF, Patzer RE, Peluso MJ, Pemu P, Pettker CM, Plunkett BA, Pogreba-Brown K, Poppas A, Quigley JG, Reddy U, Reece R, Reeder H, Reeves WB, Reiman EM, Rischard F, Rosand J, Rouse DJ, Ruff A, Saade G, Sandoval GJ, Schlater SM, Shepherd F, Sherif ZA, Simhan H, Singer NG, Skupski DW, Sowles A, Sparks JA, Sukhera FI, Taylor BS, Teunis L, Thomas RJ, Thorp JM, Thuluvath P, Ticotsky A, Tita AT, Tuttle KR, Urdaneta AE, Valdivieso D, VanWagoner TM, Vasey A, Verduzco-Gutierrez M, Wallace ZS, Ward HD, Warren DE, Weiner SJ, Welch S, Whiteheart SW, Wiley Z, Wisnivesky JP, Yee LM, Zisis S, Horwitz LI, Foulkes AS; RECOVER Consortium. Development of a Definition of Postacute Sequelae of SARS-CoV-2 Infection. JAMA. 2023 Jun;329(22):1934-46. DOI: 10.1001/jama.2023.8823

4. Darwish S, El-Boghdadly K, Edney C, Babbar A, Shembesh T. Respiratory protection in dentistry. Br Dent J. 2021 Feb;230(4):207-14. DOI: 10.1038/s41415-021-2657-0

5. Meethil AP, Saraswat S, Chaudhary PP, Dabdoub SM, Kumar PS. Sources of SARS-CoV-2 and Other Microorganisms in Dental Aerosols. J Dent Res. 2021 Jul;100(8):817-23. DOI: 10.1177/00220345211015948

6. Graziani F, Izzetti R, Lardani L, Totaro M, Baggiani A. Experimental Evaluation of Aerosol Production after Dental Ultrasonic Instrumentation: An Analysis on Fine Particulate Matter Perturbation. Int J Environ Res Public Health. 2021 Mar;18(7). DOI: 10.3390/ijerph18073357

7. Centers for Disease Control and Prevention (CDC). Interim guidance on infection control measures for 2009 H1N1 influenza in healthcare settings, including protection of healthcare personnel. Atlanta, GA: CDC; 2010 Jul 15 [accessed 2023 Oct 27]. Available from: https://www.cdc.gov/h1n1flu/guidelines_infection_control.htm.

8. Lee V, Yap J, Cook AR, Chen M, Tay J, Barr I, Kelso A, Tan B, Loh JP, Lin R, Cui L, Kelly PM, Leo Y, Chia K, Kang WL, Tambyah P, Seet B. Effectiveness of public health measures in mitigating pandemic influenza spread: a prospective sero-epidemiological cohort study. J Infect Dis. 2010 Nov;202(9):1319-26. DOI: 10.1086/656480

9. Marshall C, Kelso A, McBryde E, Barr IG, Eisen DP, Sasadeusz J, Buising K, Cheng AC, Johnson P, Richards M. Pandemic (H1N1) 2009 risk for frontline health care workers. Emerg Infect Dis. 2011 Jun;17(6):1000-6. DOI: 10.3201/eid/1706.101030

10. Yen TY, Lu CY, Chang LY, Tsai YT, Huang LM. Longitudinal seroepidemiologic study of the 2009 pandemic influenza A (H1N1) infection among health care workers in a children's hospital. BMC Infect Dis. 2012 Apr;12:89. DOI: 10.1186/1471-2334-12-89

11. Radonovich LJ Jr, Simberkoff MS, Bessesen MT, Brown AC, Cummings DAT, Gaydos CA, Los JG, Krosche AE, Gibert CL, Gorse GJ, Nyquist AC, Reich NG, Rodriguez-Barradas MC, Price CS, Perl TM; ResPECT investigators. N95 Respirators vs Medical Masks for Preventing Influenza Among Health Care Personnel: A Randomized Clinical Trial. JAMA. 2019 Sep;322(9):824-33. DOI: 10.1001/jama.2019.11645

12. Savage A. An evaluation of the impact of COVID-19 on the leadership behaviour of dental practice managers in England. BDJ Team. 2022;9:32-8. DOI: 10.1038/s41407-022-0804-3

13. Melo P, Afonso A, Monteiro L, Lopes O, Alves RC. COVID-19 Management in Clinical Dental Care Part II: Personal Protective Equipment for the Dental Care Professional. Int Dent J. 2021 Jun;71(3):263-70. DOI: 10.1016/j.identj.2021.01.007
14. Bitencourt FV, Lia EN, Pauletto P, Martins CC, Stefani CM, Massignan C, Canto GL. Prevalence of SARS-CoV-2 infection among oral health care workers worldwide: A meta-analysis. Community Dent Oral Epidemiol. 2023 Oct;51(5):718-28. DOI: 10.1111/cdoe.12827
15. Moher D, Liberati A, Tetzlaff J, Altman DG; PRISMA Group. Preferred reporting items for systematic reviews and meta-analyses: the PRISMA statement. BMJ. 2009 Jul;339:b2535. DOI: 10.1136/bmj.b2535
16. The World Bank. How does the World Bank classify countries? [accessed 2023 Nov 01]. Available from: https://datahelpdesk.worldbank.org/knowledgebase/articles/378834-how-does-the-world-bank-classify-countries
17. Munn Z, Moola S, Lisy K, Riitano D, Tufanaru C. Methodological guidance for systematic reviews of observational epidemiological studies reporting prevalence and cumulative incidence data. Int J Evid Based Healthc. 2015 Sep;13(3):147-53. DOI: 10.1097/XEB.0000000000000054
18. Munn Z, Moola S, Lisy K, Riitano D, Tufanaru C. Chapter 5: Systematic reviews of prevalence and incidence. In: Aromataris E, Munn Z, editors. JBI Manual for Evidence Synthesis. JBI; 2020 [accessed 2023 Oct 23]. DOI: 10.46658/JBIMES-20-06
19. De Sola H, Dueñas M, Salazar A, Ortega-Jiménez P, Failde I. Prevalence of Therapeutic use of Opioids in Chronic non-Cancer Pain Patients and Associated Factors: A Systematic Review and Meta-Analysis. Front Pharmacol. 2020;11:564412. DOI: 10.3389/fphar.2020.564412
20. Abo-Leyah H, Gallant S, Cassidy D, Giam YH, Killick J, Marshall B, Hay G, Snowdon C, Hothersall EJ, Pembridge T, Strachan R, Gallant N, Parcell BJ, George J, Furrie E, Chalmers JD. The protective effect of SARS-CoV-2 antibodies in Scottish healthcare workers. ERJ Open Res. 2021 Apr;7(2). DOI: 10.1183/23120541.00080-2021
21. Abu-Hammad O, Alnazzawi A, Babkair H, Jambi S, Mirah M, Abdouh I, Aljohani RS, Ayeq R, Ghazi L, Al-Subhi H, Dar-Odeh N. COVID-19 Infection in Academic Dental Hospital Personnel; A Cross-Sectional Survey in Saudi Arabia. Int J Environ Res Public Health. 2021 Oct;18(20). DOI: 10.3390/ijerph182010911
22. Akbari N, Salehiniya H, Abbaszadeh H. The prevalence of COVID-19 in dentists and dental assistants. J Biostat Epidemiol. 2021; 7:174-84. DOI: 10.18502/jbe.v7i2.6726
23. Al-Kuwari MG, AbdulMalik MA, Al-Nuaimi AA, Abdulmajeed J, Al-Romaihi HE, Semaan S, Kandy M. Epidemiology Characteristics of COVID-19 Infection Amongst Primary Health Care Workers in Qatar: March-October 2020. Front Public Health. 2021;9:679254. DOI: 10.3389/fpubh.2021.679254
24. Antonio-Villa NE, Bello-Chavolla OY, Vargas-Vázquez A, Fermín-Martínez CA, Márquez-Salinas A, Pisanty-Alatorre J, Bahena-López JP. Assessing the Burden of Coronavirus Disease 2019 (COVID-19) Among Healthcare Workers in Mexico City: A Data-Driven Call to Action. Clin Infect Dis. 2021 Jul;73(1):e191-e198. DOI: 10.1093/cid/ciaa1487
25. Araujo MWB, Estrich CG, Mikkelsen M, Morrissey R, Harrison B, Geisinger ML, Ioannidou E, Vujicic M. COVID-2019 among dentists in the United States: A 6-month longitudinal report of accumulative prevalence and incidence. J Am Dent Assoc. 2021 Jun;152(6):425-33. DOI: 10.1016/j.adaj.2021.03.021
26. Bontà G, Campus G, Cagetti MG. COVID-19 pandemic and dental hygienists in Italy: a questionnaire survey. BMC Health Serv Res. 2020 Oct;20(1):994. DOI: 10.1186/s12913-020-05842-x
27. Cintora P, Rojo R, Martínez A, Ruíz B, Aragoneses JM. Seroprevalence of SARS-CoV-2 in a fully operative dentistry academic center in Madrid (Spain) during the de-escalation phase of the COVID-19 pandemic. Are our dentists at greater risk? Oral Health Prev Dent. 2022; 20:349-53.
28. Duś-Ilnicka I, Szczygielska A, Kuźniarski A, Szymczak A, Pawlik-Sobecka L, Radwan-Oczko M. SARS-CoV-2 IgG Amongst Dental Workers During the COVID-19 Pandemic. Int Dent J. 2022 Jun;72(3):353-9. DOI: 10.1016/j.identj.2022.02.003
29. Estrich CG, Gurenlian JR, Battrell A, Bessner SK, Lynch A, Mikkelsen M, et al. COVID-19 prevalence and related practices among dental hygienists in the United States. J Dent Hyg. 2021; 95:6-16.
30. Ferreira RC, Gomes VE, Rocha NBD, Rodrigues LG, Amaral JHLD, Senna MIB, Alencar GP. COVID-19 Morbidity Among Oral Health Professionals in Brazil. Int Dent J. 2022 Apr;72(2):223-9. DOI: 10.1016/j.identj.2021.05.005

31. Fredriksson L, Cederlund A, Murray M, Jansson L, Skott P. Prevalence of ongoing or previous SARS-CoV-2 infection among dental personnel - the Swedish experience. Acta Odontol Scand. 2023 Mar;81(2):119-23. DOI: 10.1080/00016357.2022.2095023
32. Froum SH, Froum SJ. Incidence of COVID-19 Virus Transmission in Three Dental Offices: A 6-Month Retrospective Study. Int J Periodontics Restorative Dent. 2020;40(6):853-9. DOI: 10.11607/prd.5455
33. Gallus S, Paroni L, Re D, Aiuto R, Battaglia DM, Crippa R, Carugo N, Beretta M, Balsano L, Paglia L. SARS-CoV-2 Infection among the Dental Staff from Lombardy Region, Italy. Int J Environ Res Public Health. 2021 Apr;18(7). DOI: 10.3390/ijerph18073711
34. Hosoglu S, Mahmood MK. COVID-19 infection among dentists in Iraqi Kurdistan Region. J Infect Dev Ctries. 2022 Sep;16(9):1439-44. DOI: 10.3855/jidc.15962
35. Jungo S, Moreau N, Mazevet ME, Ejeil AL, Biosse Duplan M, Salmon B, Smail-Faugeron V. Prevalence and risk indicators of first-wave COVID-19 among oral health-care workers: A French epidemiological survey. PLoS One. 2021;16(2):e0246586. DOI: 10.1371/journal.pone.0246586
36. Lucaciu O, Boca A, Mesaros AS, Petrescu N, Aghiorghiesei O, Mirica IC, Hosu I, Armencea G, Bran S, Dinu CM. Assessing SARS-CoV-2 Infection Rate among Romanian Dental Practitioners. Int J Environ Res Public Health. 2021 May;18(9). DOI: 10.3390/ijerph18094897
37. Madathil S, Siqueira WL, Marin LM, Sanaulla FB, Faraj N, Quiñonez CR, McNally M, Glogauer M, Allison P. The incidence of COVID-19 among dentists practicing in the community in Canada: A prospective cohort study over a 6-month period. J Am Dent Assoc. 2022 May;153(5):450-9.e1. DOI: 10.1016/j.adaj.2021.10.006
38. Mksoud M, Ittermann T, Holtfreter B, Söhnel A, Söhnel C, Welk A, Ulm L, Becker K, Hübner NO, Rau A, Kindler S, Kocher T. Prevalence of SARS-CoV-2 IgG antibodies among dental teams in Germany. Clin Oral Investig. 2022 May;26(5):3965-74. DOI: 10.1007/s00784-021-04363-z
39. Molvik M, Danielsen AS, Grøsland M, Telle KE, Kacelnik O, Eriksen-Volle HM. SARS-CoV-2 in health and care staff in Norway, 2020. Tidsskr Nor Laegeforen; 2021. p. 141.
40. Moraes RR, Correa MB, Martins-Filho PR, Lima GS, Demarco FF. COVID-19 incidence, severity, medication use, and vaccination among dentists: survey during the second wave in Brazil. J Appl Oral Sci. 2022;30:e20220016. DOI: 10.1590/1678-7757-2022-0016
41. Ribeiro JAM, Farias SJS, Souza TAC, Stefani CM, Lima AA, Lia EN. SARS-CoV-2 infection among Brazilian dentists: a seroprevalence study. Braz Oral Res. 2022;36:e035. DOI: 10.1590/1807-3107bor-2022.vol36.0035
42. Rock LD, Madathil S, Khanna M, Macdonald LK, Quiñonez C, Glogauer M, et al. COVID-19 incidence and vaccination rates among Canadian dental hygienists. Can J Dent Hyg. 2022; 56:123-30.
43. Santana LADM, Pinho JNA, de Albuquerque HIM, Souza LMA. COVID-19 contamination among maxillofacial surgeons and impact in Brazilian public center. J Stomatol Oral Maxillofac Surg. 2022 Apr;123(2):92-94. DOI: 10.1016/j.jormas.2021.05.001
44. Sarapultseva M, Hu D, Sarapultsev A. SARS-CoV-2 Seropositivity among Dental Staff and the Role of Aspirating Systems. JDR Clin Trans Res. 2021 Apr;6(2):132-8. DOI: 10.1177/2380084421993099
45. Schmidt J, Perina V, Treglerova J, Pilbauerova N, Suchanek J, Smucler R. COVID-19 Prevalence among Czech Dentists. Int J Environ Res Public Health. 2021 Nov;18(23). DOI: 10.3390/ijerph182312488
46. Puia S, Pasart J, Gualtieri A, Somoza F, Melo C, Alessandrelo M, Gatti P, Squassi A, Rodriguez PA. Corrigendum to “Assesment of SARS-CoV-2 infection-in dentists and supporting staff at a university dental hospital in Argentina”. Journal of Oral Biology and Craniofacial Research Volume 11, Issue 2 (2021) Pages 169-173. J Oral Biol Craniofac Res. 2021;11(4):659. DOI: 10.1016/j.jobcr.2021.09.013
47. Shields AM, Faustini SE, Kristunas CA, Cook AM, Backhouse C, Dunbar L, Ebanks D, Emmanuel B, Crouch E, Kröger A, Hirschfeld J, Sharma P, Jaffery R, Nowak S, Gee S, Drayson MT, Richter AG, Dietrich T, Chapple ILC. COVID-19: Seroprevalence and Vaccine Responses in UK Dental Care Professionals. J Dent Res. 2021 Oct;100(11):1220-7. DOI: 10.1177/00220345211020270
48. Suarez-Cabello C, Valdivia E, Vergara-Buenaventura A. Clinical-Epidemiological Profile of Dental Professionals Associated with COVID-19 Infection in Southern Peru: A Cross-Sectional Study. Int J Environ Res Public Health. 2022 Dec;20(1). DOI: 10.3390/ijerph20010672

49. Cagetti MG, Cairoli JL, Senna A, Campus G. COVID-19 Outbreak in North Italy: An Overview on Dentistry. A Questionnaire Survey. Int J Environ Res Public Health. 2020 May;17(11). DOI: 10.3390/ijerph17113835

50. World Health Organization Writing GroupBell D, Nicoll A, Fukuda K, Horby P, Monto A, Hayden F, Wylks C, Sanders L, Van Tam J. Non-pharmaceutical interventions for pandemic influenza, international measures. Emerg Infect Dis. 2006 Jan;12(1):81-7. DOI: 10.3201/eid1201.051370

51. Lerche N, Holtfreter S, Walther B, Semmler T, Al'Sholui F, Dancer SJ, Daeschlein G, Hübner NO, Bröker BM, Papke R, Kohlmann T, Baguhl R, Seifert U, Kramer A. Staphylococcus aureus nasal colonization among dental health care workers in Northern Germany (StaphDent study). Int J Med Microbiol. 2021 Aug;311(6):151524. DOI: 10.1016/j.ijmm.2021.151524

52. Brito-Reia VC, da Silva Bastos R, Vieira Vilhena F, Marques Honório H, Marques da Costa Alves L, Frazão P, Sérgio da Silva Santos P. Population-based virucidal phthalocyanine gargling/rinsing protocol to reduce the risk of coronavirus disease-2019: a community trial. GMS Hyg Infect Control. 2022 Dec 6;17:Doc23. DOI: 10.3205/dgkh000426

53. Kramer A, Eggers M, Exner M, Hübner NO, Simon A, Steinmann E, Walger P, Zwicker P. Recommendation of the German Society of Hospital Hygiene (DGKH): Prevention of COVID-19 by virucidal gargling and virucidal nasal spray - updated version April 2022. GMS Hyg Infect Control. 2022 Jul 7;17:Doc13. DOI: 10.3205/dgkh000416

54. Kramer A, Eggers M, Hübner NO, Walger P, Steinmann E, Exner M. Virucidal gargling and virucidal nasal spray. GMS Hyg Infect Control. 2021 Jan 18;16:Doc02. DOI: 10.3205/ dgkh000373

55. Lenharo M. WHO declares end to COVID-19's emergency phase. Nature. 2023 May 5. DOI: 10.1038/d41586-023-01559-z

56. European Centre for Disease Prevention and Control (ECDC). SARS-CoV-2 variants of concern as of 20 October 2023. 2023 [accessed 2023 Oct 23]. Available from: https://www.ecdc.europa.eu/en/covid-19/variants-concern

57. World Health Organization. From emergency response to long-term COVID-19 disease management: sustaining gains made during the COVID-19 pandemic.Geneva: WHO; 2023 May 03.

58. World Health Organization. WHO Coronavirus (COVID-19) Dashboard. [accessed 2023 Oct 26]. Available from: https://covid19.who.int/

59. Lippi G, Mattiuzzi C, Henry BM. Uncontrolled confounding in COVID-19 epidemiology. Diagnosis (Berl). 2023 May;10(2):200-2. DOI: 10.1515/dx-2022-0128

60. Bundesministerium für Gesundheit. Corona-Testverordnung. [accessed 2023 Nov 02]. Available from: https://www.bundesgesundheitsministerium.de/coronavirus/nationale-teststrategie/coronavirus-testver-ordnung.html

61. ECDC-EMA. ECDC-EM statement on updating COVID-19 vaccines composition for new SARS-CoV-2 virus variants. EMA/257222/2023. European Medicines Agency; 2023 Jun 06

62. European Centre for Disease Prevention and Control (ECDC]. Country overview report: week 40 2023. 2023 Oct 25 [accessed 2023 Nov 02]. Available from: https://www.ecdc.europa.eu/en/covid-19/country-overviews
