# Supplementary material for: Risk of SARS-CoV-2 infection in dental healthcare workers – a systematic review and meta-analysis
Source: GMS Hyg Infect Control. 2024 Mar 5;19:Doc09. doi: 10.3205/dgkh000464 (PMC11035909; doi:10.3205/dgkh000464)
Supplement: Table S2: Risk assessement [file HIC-19-09-s-002.pdf]

Attachment 2

Table S2: Risk assessement

Author, publication year, country	Comparison groups	SARS-CoV-2 prevalence/ incidence	Implementation of personal protective measures (PPE)	Comparison with subgroups or the general population	Potential bias/ limitations	Discussion
Abo-Leyah H. et al., 2020, UK [20]	DCW; Other HCW; General Scottish population (GP)	Prevalence in DCW was 26% (n=13/50)	No information on the use of PPE in dentists. According to the authors, In critical care areas all staff should have worn PPE in accordance with Health Protection Scotland guidance on working in aerosol-generating procedures. Therefore, there, seroprevalence was only 16% (n=21/31).	No statistically significant difference in prevalence between dentists and other HCW (recalu-lated OR 1.82 [95% CI, 0.98-3.40]). SARS-CoV-2 infections in dentists >3 times higher than in the GP (4.5%; no absolute numbers provided)	Selection bias possible	Measures to protect even high-risk front-line staff appeared to be insufficient
Abu-Hammad O, et al., 2021, Saudi Arabia [21]	None	COVID-19 prevalence of 19.6% (n=62/316)	No information on supply with and use of PPE	10 cases did not wear face masks; no quarantine in 7 of the infected persons	-	Infection control insufficient at that time
Akbari N, et al., 2021, Iran [22]	Dentists; Dentists' assistants	Prevalence in DCW 0.4% (n=3/762)	No information on supply with and use of PPE	Prevalence 0.78% (n=3/381) in dentists tested by "paraclinical" tests"; 0% (n=0/381) in dentists' assistants (difference ns)	Selection bias possible	Overall prevalence of COIVD-19 in dentists and their assistants very low. According to the authors use of standard PPE is sufficient to prevent SARS-CoV-2 infections.
Al Kuwari M, et al., 2021, Qatar [23]	Subgroups of primary HCW (e.g. nurses, physicians, pharmacists, lab technicians, dentists, dental staff, radiology staff)	Total prevalence in the 7407 staff members who underwent COVID-19 RT-PCR testing was 16.2% (n=1199/7407)	No infirmation on the actual use of PPE in the compared subgroups	Difference in prevalence of dentists [11.2% (n=22/196)] and dental staff members [7.3% (n=14/192)] ns. Significantly lower prevalence in DCW compared to that in the staff members of other subgroups (calculated OR 0.55; 95% CI, 0.39-0.77; P <0.001)	Selection bias possible as it remains unclear to which subgroups the missing 19% of employees members can be assigned.	Lower SARS-CoV-2 prevalence in dental HCW may be attributed to more proper usage of PPE.

Author, publication year, country	Comparison groups	SARS-CoV-2 prevalence/ incidence	Implementation of personal protective measures (PPE)	Comparison with subgroups or the general population	Potential bias/ limitations	Discussion
Antonio-Villa MD, et al., 2021, Mexico [24]	All HCW, dentists; General population in Mexico City	Prevalence in dentists (extrapolated) 25.3% (n=267/1054)	No mentioning of the availability of or adherence to PPE	Dentists had a significantly lower prevalence (P <0.001) than “all” HCW with 30.3% (n=17531/57758), but a higher prevalence than in the general population in Mexico City with 4.61% (n=403,185/8,737,172).	-	HCWs have increased occupational hazard to acquire SARS-CoV-2 infection compared with the general population, attributable to direct contact during care of hospitalized patients.
Araujo M, et al., 2021, USA [25]	None	Cumulative COVID-19 infection prevalence rate (n=57/2196) was 2.6%; incidence ranging between 0.2% and 1.1% each month	Statistically significant decline in dentists reporting sometimes or always wearing N95 or equivalent masks and eye protection during AGPs over time from 92.4% in the first survey to 88.0% in the final survey (P <0.01)	-		Despite of a minor shift in the use of PPE during the 6-month period of the study, US dentists show a high level of adherence to enhanced infection control procedures.
Bonta G, et al., 2020, Italy [26]	Dental hygienists clustered in 3 groups (1-10, 11-20 and 20+ professional experience)	0.25% (n=7/2798) reported COVID-19 infections.	82.8% of dental hygienists wore surgical masks and 90.55% protective glasses or visors.	No statistical difference regarding prevalence between the three group	Response bias possible	This data might suggest a low infection rate among dental hygienists, just as the appropriate preventive measures were correctly implemented.
Cintora P, et al., 2022, Spain [27]	Internal comparison between dental subdisciplines taking administrative staff as reference	Prevalence among all participants 20% (n=39/195)	No information on the use of PPE provided	Prevalence in doctors 23.95% (n=37/155) compared to 5% (n=2/40) in administrative staff (OR 5.96 [95% CI, 1.4-25.9]). Seroprevalence in orthodontists was 34.8% (n=24/69), followed by the pediatric dentists [28.6% (n=2/7)], oral surgeons [14.7% (n=10/68)] and endodontists [9.1% (n=1/11)]. In multivariate analysis only orthodontists have a significantly higher chance of becoming Infected: OR [10.13 (95% CI, 2.25-45.68)].	-	Covid-19 is more prevalent in orthodontists than in the rest of dental subdisciplines with the lowest rate among the administrative staff. This result puts orthodontics on alert regarding the risk of COVID-19 transmission.
Dus-Ilnicka I, et al., 2022, Poland [28]	Internal comparison of different subgroups with each other (e.g., dental surgeons, orthodontists)	8 (6.3%) of all volunteers had positive results for SARS-CoV-2 IgG antibodies.	Numerous protective measures were reinforced (e.g. phone-only registration and gravitational ventilation) but the use of face masks is not mentioned.	Difference of prevalences between SUB1: n=4/67 (6%); SUB2: n=3/40 (7.5%); SUB3; n=1/20 (5%) not statistically significant.	Selection bias (the survey did not cover all the DCW from the Academic Dental Polyclinic [180 dental workers in total?])	According to the authors the risk of COVID-19 transmission in dental offices is low if safety measures are followed.

Author, publication year, country	Comparison groups	SARS-CoV-2 prevalence/ incidence	Implementation of personal protective measures (PPE)	Comparison with subgroups or the general population	Potential bias/ limitations	Discussion
Estrich GC, et al., 2021, USA [29]	Dental hygienists; General US population (as of 8 October 2020 an estimated 2.3% or 7.6 million people have had COVID-19)	3.1% (n=149/4776) had ever tested positive or been diagnosed with COVID-19	Only 55.7% (n=1871/3357) always used PPE in the past month. Over half (54.6%, n=659) of those with 10 or less years of experience always used PPE according to CDC guidelines, compared with 55.4% (n=511) of those with 11-20 years and 60.7% (n=692) of those with 21 or more years of experience (P <0.01).	As of October 2020, the estimated cumulative COVID-19 prevalence in dental hygienists in the US was low, but higher than found in the general US population	Response bias; study based on self-reported data	Years of experience as a dental hygienist was significantly associated with always following CDC PPE guidelines.
Ferreira RC, et al., 2021, Brazil [30]	Dental HCW; General Brazil population	Total prevalence among dental HCW 21.68% (n= 10,473/ 48,301). Age-standardized cumulative incidence 17.7/1000 (no 95% CI provided)	No information on the actual use of PPE	SARS-CoV-2 infections more frequent compared to the general population: Age-standardized cumulative incidence 18.7/1000 for dental HCW and 17.71/1000 for the general population (ratio 1.05) Subgroup analysis: Prevalence in dentists 21.19% (n=6710/31,666); in other DCW 22.6% (n=3763/16,635); difference in χ^2 test ns.	Selection bias possible because only 72.26% of data of health professionals were analyzed (no data for dental HCW reported).	In light of the 5% higher cumulative incidence of infections among DCW compared to the general population the authors highlight the importance of PPE in dental practice.
Fredriksson L, et al., 2023, Sweden [31]	Clinical and administrative dental employees; General population (seroprevalence in inhabitants of Stockholm in June 2020 11.7%)	Prevalence 12.3% (n=42/341 SARS-CoV-2 infections verified by RT-PCR or antibodies)	No figures on supply with or adherence to PPE provided	No separate prevalence figures available for doctors and assistant DCW. No statistically significant difference in prevalence between clinical (n=197/337; 58.5%) and administrative employees (n=140/337; 58.5%); also no significant difference with respect to patient-related work: daily (42.6%); sporadically (25.8%); never (31.5%)	Selection bias; type of randomization not revealed, no sample size calculation	Prevalence in the selected group of dental health workers did not differ from that of the general population. Lack of statistical difference in prevalence regarding patient contact suggests that non-clinical SARS-CoV-2 infections originated mainly outside the clinical activities.

Author, publication year, country	Comparison groups	SARS-CoV-2 prevalence/ incidence	Implementation of personal protective measures (PPE)	Comparison with subgroups or the general population	Potential bias/ limitations	Discussion
Froum SJ, et al., 2020, USA [32]	None	No COVID-19 infection in dental health workers working in the offices during the observation period	Wearing of FFP2 masks and maximum protection mandatory (e.g., hand washing, HEPAC air filters and UV-C germicidal lights)	Not applicable	No inclusion in the prevalence calculation possible because a denominator (number of employees at risk during the observation period) is missing.	Dental health care can be administered safely even when exposed to high risk patients.
Gallus S, et al., 2021, Italy [33]	Internal comparison between different groups (dentists, dental technicians; dental assistants, administrative personnel; students and others)	10.8% HCW (n=54/499) tested positive; of those 10.9% dentists (n=20/183), 13.0% dental technicians (n=3/23), 7.1% dental hygienists (n=2/28) and 8.4% dental assistants (n=15/179)	It is stated that dentists are able to protect themselves by utilizing PPE under the premise that they had already worked for several decades. However, the actual use of "protective devices" was not queried.	No statistically significant difference in prevalence between any group.	-	The clinical staff was less frequently tested positive to SARS-CoV-2 than the administrative staff probably due to more scrupulous management of protective devices.
Hosoglu S, et al., 2022, Iraq [34]	Impact of 13 main variables associated with SARS-CoV-2 infection in dentists investigated	25.3% (n=21/83) stated a positive PCR.	PPE was always available for only 50.6% of the participating dentists. No information on wearing of masks available; only information on wearing glasses (83.6%) and hand washings (91.6%) found	Working in a public hospital COVID 19 in dentists (n=12/14) was a risk factor for infection (OR 6.8 [95% CI, 1.2-37.6)].<br>In univariate analysis, paradoxically, a lower working time was associated with SARS-CoV-2 positivity (17.3 ± 11.0 hr versus 23.8 ± 13.7 hr; P =0.020)	-	Insufficient prevention measures may have contributed to the high prevalence of infection among dentists.
Jungo S, et al., 2021, France [35]	Dentists; dental assistants; General French population at the same date (2% COVID-19 infections)	Prevalence of laboratory-confirmed COVID-19 was 1.54% (n=93/6040).	In the subgroup of symptomatic dentists (n=1097) and dental assistants (n=360), dental assistants used FFP2 masks and safety goggles less frequently than dentists (3.9% vs 8.8% and 39.2% vs 62%, respectively (P <0.01 and P <0.001).	Prevalence in dentists with 1.9% (n=79/4172) was significantly higher than in dental assistants with 0.8% (n=14/1868); calculated OR 2.56 (95% CI, 1.44-4.52).	Possible selection bias	According to the authors healthcare professionals did not have a higher risk of COVID-19 overall than the general population.
Lucaciu O, et al., 2021, Romania [36]	Dentists; dental assistants; General Romanian population	The calculated infection rate for the period between March 2020 and March 2021 was 6.37% (n=238/3735)	No information on the extent of implementing PPE in individual employees or number of dental offices.	6.46% (117/1811) in dentists and 6.37% (121/1924) in dental assistants (difference ns). Prevalence in the general Romanian population 0.26% (March 2020 to February 2021)	Possible response bias as the total number of invited offices not reported.	According to the authors most infections ("contaminations") occurred outside the dental offices. Protective measures not sufficiently implemented at that time.

Author, publication year, country	Comparison groups	SARS-CoV-2 prevalence/ incidence	Implementation of personal protective measures (PPE)	Comparison with subgroups or the general population	Potential bias/ limitations	Discussion
Madathil S, et al., 2022, Canada [37]	Dentists; General population	Prevalence 4.17% (6/644). Incidence rate 5.1 per 100,000 person-days (95%CI, 1.86-9.91)	Whilst the routine use of surgical masks decreased form 91.8% to 79.4 during the follow-up period (every four weeks), the use of N95 respirators was low increasing from 40.2% over time to 62.9% at the end of the follow up.	Incidence proportion 1084 per 100,000 dentists (95% CI, 438 to 2011 per 100,000 dentists); incidence proportion in the general population 1864 per 100.000 people [95% CI, 1.859 to 1.868)	-	Less COVID-19 infections observed among Canadian dentists compared to that of the general population
Mksoud M, et al., 2022, Germany [38]	Dentists (n=927); dental nurses and prophylaxis nurses; (n=1812); General German population	Prevalence 6.43% (n=179/2784) [IgG antibodies and/or positive SARS-Cov-2 PCR test]	74.2% FFP masks; 63.9% visors, 77.7% safety goggles; 12.6% rubber dams; mean number of protective measures 2.6 (± 1.2 SD)	Prevalence in dentists 5.5% (n=53/972) and 7.0% (n=126/1812) in other dental HCWs; difference ns.<br>No single protective measure (e.g., FFP mask, use of rubber dam, number of aerosol-generating devices) significantly associated with SARS-CoV-2 positivity.<br>7% IgG antibodies were reported in March 2021 across Germany.	Selection bias possible	According to the authors the prevalence of SARS-CoV-2 antibodies in dental team members is comparable to that of the general population.
Molvik M, et al., 2021, Norway [39]	Dentists; other Norwegian HCW	Prevalence among dentists 1.19% (n=35/2941)	No information on the number of HCW wearing PPE	Prevalence in all HCW 1.48% (n=5673/382,332); difference to prevalence in dentists ns.	Selection bias (no self-employed HCW included)	Incidence rates in the first 4 weeks may be under-estimated, as test data are incomplete before 1 April 2020.
Moraes RF, et al., 2022, Brazil [40]	Dentists; General Brazil population	26.6% (n=466/1754) of participants had tested positive for SARS-CoV-2 by May 2021	Only 69.1 (n=1182/1710) dentists always wearing N95 masks in appointment with patients and only 59.6% (n=1012/1699) wearing face shields.	Prevalence in dentists higher than that in the general Brazilian population (7.9% confirmed SARS-CoV-2 infections by May 2021)	-	High prevalence of COVID-19 infections among Brazilian dental professionals
Ribeiro JM, et al., 2021, Brazil [41]	Dentists; General population of the Federal district	Seroprevalence 19.1% (n=62/324)	No information on supply with and adherence to PPE	Prevalence associated only with COVID-19 in a household member (OR 2.5, [95% CI, 1.13–5.3]) and treatment of patients with fever (OR 2.99 [95% CI, 1.03–8.70]); results similar to that of the general population<br>(17% of 1077 tested residents SARS-CoV-2 positive by December 2020)	-	The consistent use of personal protection measures are doubted by the authors as dentists were considered more rigorous in using PPE when treating patients with fever.

Author, publication year, country	Comparison groups	SARS-CoV-2 prevalence/ incidence	Implementation of personal protective measures (PPE)	Comparison with subgroups or the general population	Potential bias/ limitations	Discussion
Rock LD, et al., 2022, Canada [42]	Dentists; General population in the correspon-ing Canadian provinces	20 participants infected during the follow-up period (cumulative incidence 2.39% (95% CI 1.49%–3.50%)	No concrete information on PPE. According to the authors the low infection rate is a cumulative effect of "all the different protective measures" that have been employed.	Cumulative incidence lower than that in the general population (5.12% [95% CI, 5.12%–5.13%]) during the same period. 69% of participants had received two vaccine doses, and 19.7% three vaccine doses.	Response bias	Beside other protective measures, uptake of vaccination may explain low COVID-19 infection rates.
Santana LADM, et al., 2021, Brazil [43]	None	75.2% (n=15/20) tested positive (RT-PCR or antibodies) including 4 cases of reinfection	No information on use of PPE	No other DCW than surgeons tested for SARS-CoV-2	Arbitrarily selected observation	Shortfall of PPE, inadequate decontamination and sharing of the emergency room with other healthcare professionals and their patients contributed to high rate of COVID-19 infections
Sarapultseva M, et al., 2021, Russia [44]	Dentists; Dental assistants; General population	12.1% (n=19/157)	No information on use of PPE	Dentists 10.26% (n=8/78); dental assistant 13.92% (n=11/79); difference ns (prevalence unaffected by sex or role of the member in the dental team). Seroprevalence rate in the general population of the Russian Federation 7.4-9.3%	Arbitrarily selected observation	SARS-CoV-2 infection in DCW higher than in the Russian population, according to the authors due the increased risk.
Schmidt J, et al., 2021, Czechia [45]	Dentists; Czech general population	13.9% PCR-positive (n=377/2716)	No information on supply with and adherence to PPE	Prevalence significantly lower than that of the general population (13.9% vs 15.64%; P =0.018)	Underreporting bias (incomplete information on actual test positivity as 154 respondents with clinical symptoms had not been tested and were not considered in the evaluation).	Proper focus on infection control led to a reduction in occupational infection risks.
Sebastian P, et al., 2021, Argentina [46]	None	Prevalence 4.47% (n=16/358; 95% CI, 3-7%)	PPE was used in all work-related tasks.	No differentiation between dentists and assistant dental workers made. No significant difference in test results between clinical or non-clinical job type	-	Low rates of COVID-19 infection among clinical personnel may be due to proper use of PPE

Author, publication year, country	Comparison groups	SARS-CoV-2 prevalence/ incidence	Implementation of personal protective measures (PPE)	Comparison with subgroups or the general population	Potential bias/ limitations	Discussion
Shields AM, et al., 2021, UK [47]	Dentists; other DCW; General population in the West Midlands region	Prevalence of spike antibodies at baseline 16.3% (n=246/1507)	According to the authors dentistry reopened until July 2020 "including FFP3 masks, eye protection, and gowns". No information on the actual supply with and adherence to PPE	Prevalence in dentists 16.7% (115/687); other DCW 16.0% (131/820), difference ns. Seroprevalence in DCW higher than that of the general population seroprevalence at the time of baseline sampling (6-7%)	-	Initial use of PPE was not sufficient in reducing the risk of occupational exposure. The risk of PCR-proven infection in seronegative DCW decreased between June 2020 and January 2021 to 11.7% suggesting enhanced infection control, including FFP3 masks.
Suarez-Cabello C, et al., 2022, Peru [48]	None	Prevalence 43.9% (n=179/408)	No information on supply with and adherence to PPE	Job context unclear as only 357/408 (87.5%) participants had worked during the pandemic	Selection bias possible	There is a likelihood that COVID-19-transmissions may have occurred in non-dental settings.

AGP: aerosol generating procedures

DCW: dental healthcare workers

FFP: filtering face piece

GDP: general dental practitioner

GP: general population

HCW: healthcare workers

hr: hours

ns: not significant

OR: odds ratio

PPE: personal protective equipment

RT-PCR: real-time PCR test

SD: standard deviation

vs: versus

yr: years

References

1. WHO. Coronavirus disease (COVID-19). Geneva: World Health Organization; 2023 [accessed 2023 Oct 26]. Available from: https://www.who.int/emergencies/diseases/novel-coronavirus-2019
2. Global tuberculosis report 2022. Geneva: World Health organization; 2022. p. 68.
3. Thaweethai T, Jolley SE, Karlson EW, Levitan EB, Levy B, McComsey GA, McCorkell L, Nadkarni GN, Parthasarathy S, Singh U, Walker TA, Selvaggi CA, Shinnick DJ, Schulte CCM, Atchley-Challenner R, Alba GA, Alicic R, Altman N, Anglin K, Argueta U, Ashktorab H, Baslet G, Bassett IV, Bateman L, Bedi B, Bhattacharyya S, Bind MA, Blomkalns AL, Bonilla H, Bush PA, Castro M, Chan J, Charney AW, Chen P, Chibnik LB, Chu HY, Clifton RG, Costantine MM, Cribbs SK, Davila Nieves SI, Deeks SG, Duven A, Emery IF, Erdmann N, Erlandson KM, Ernst KC, Farah-Abraham R, Farner CE, Feuerriegel EM, Fleurimont J, Fonseca V, Franko N, Gainer V, Gander JC, Gardner EM, Geng LN, Gibson KS, Go M, Goldman JD, Grebe H, Greenway FL, Habli M, Hafner J, Han JE, Hanson KA, Heath J, Hernandez C, Hess R, Hodder SL, Hoffman MK, Hoover SE, Huang B, Hughes BL, Jagannathan P, John J, Jordan MR, Katz SD, Kaufman ES, Kelly JD, Kelly SW, Kemp MM, Kirwan JP, Klein JD, Knox KS, Krishnan JA, Kumar A, Laiyemo AO, Lambert AA, Lanca M, Lee-Iannotti JK, Logarbo BP, Longo MT, Luciano CA, Lutrick K, Maley JH, Marathe JG, Marconi V, Marshall GD, Martin CF, Matusov Y, Mehari A, Mendez-Figueroa H, Mermelstein R, Metz TD, Morse R, Mosier J, Mouchati C, Mullington J, Murphy SN, Neuman RB, Nikolich JZ, Ofotokun I, Ojemakinde E, Palatnik A, Palomares K, Parimon T, Parry S, Patterson JE, Patterson TF, Patzer RE, Peluso MJ, Pemu P, Pettker CM, Plunkett BA, Pogreba-Brown K, Poppas A, Quigley JG, Reddy U, Reece R, Reeder H, Reeves WB, Reiman EM, Rischard F, Rosand J, Rouse DJ, Ruff A, Saade G, Sandoval GJ, Schlater SM, Shepherd F, Sherif ZA, Simhan H, Singer NG, Skupski DW, Sowles A, Sparks JA, Sukhera FI, Taylor BS, Teunis L, Thomas RJ, Thorp JM, Thuluvath P, Ticotsky A, Tita AT, Tuttle KR, Urdaneta AE, Valdivieso D, VanWagoner TM, Vasey A, Verduzco-Gutierrez M, Wallace ZS, Ward HD, Warren DE, Weiner SJ, Welch S, Whiteheart SW, Wiley Z, Wisnivesky JP, Yee LM, Zisis S, Horwitz LI, Foulkes AS; RECOVER Consortium. Development of a Definition of Postacute Sequelae of SARS-CoV-2 Infection. JAMA. 2023 Jun;329(22):1934-46. DOI: 10.1001/jama.2023.8823
4. Darwish S, El-Boghdadly K, Edney C, Babbar A, Shembesh T. Respiratory protection in dentistry. Br Dent J. 2021 Feb;230(4):207-14. DOI: 10.1038/s41415-021-2657-0
5. Meethil AP, Saraswat S, Chaudhary PP, Dabdoub SM, Kumar PS. Sources of SARS-CoV-2 and Other Microorganisms in Dental Aerosols. J Dent Res. 2021 Jul;100(8):817-23. DOI: 10.1177/00220345211015948
6. Graziani F, Izzetti R, Lardani L, Totaro M, Baggiani A. Experimental Evaluation of Aerosol Production after Dental Ultrasonic Instrumentation: An Analysis on Fine Particulate Matter Perturbation. Int J Environ Res Public Health. 2021 Mar;18(7). DOI: 10.3390/ijerph18073357
7. Centers for Disease Control and Prevention (CDC). Interim guidance on infection control measures for 2009 H1N1 influenza in healthcare settings, including protection of healthcare personnel. Atlanta, GA: CDC; 2010 Jul 15 [accessed 2023 Oct 27]. Available from: https://www.cdc.gov/h1n1flu/guidelines_infection_control.htm.
8. Lee V, Yap J, Cook AR, Chen M, Tay J, Barr I, Kelso A, Tan B, Loh JP, Lin R, Cui L, Kelly PM, Leo Y, Chia K, Kang WL, Tambyah P, Seet B. Effectiveness of public health measures in mitigating pandemic influenza spread: a prospective sero-epidemiological cohort study. J Infect Dis. 2010 Nov;202(9):1319-26. DOI: 10.1086/656480
9. Marshall C, Kelso A, McBryde E, Barr IG, Eisen DP, Sasadeusz J, Buising K, Cheng AC, Johnson P, Richards M. Pandemic (H1N1) 2009 risk for frontline health care workers. Emerg Infect Dis. 2011 Jun;17(6):1000-6. DOI: 10.3201/eid/1706.101030
10. Yen TY, Lu CY, Chang LY, Tsai YT, Huang LM. Longitudinal seroepidemiologic study of the 2009 pandemic influenza A (H1N1) infection among health care workers in a children's hospital. BMC Infect Dis. 2012 Apr;12:89. DOI: 10.1186/1471-2334-12-89
11. Radonovich LJ Jr, Simberkoff MS, Bessesen MT, Brown AC, Cummings DAT, Gaydos CA, Los JG, Krosche AE, Gibert CL, Gorse GJ, Nyquist AC, Reich NG, Rodriguez-Barradas MC, Price CS, Perl TM; ResPECT investigators. N95 Respirators vs Medical Masks for Preventing Influenza Among Health Care Personnel: A Randomized Clinical Trial. JAMA. 2019 Sep;322(9):824-33. DOI: 10.1001/jama.2019.11645
12. Savage A. An evaluation of the impact of COVID-19 on the leadership behaviour of dental practice managers in England. BDJ Team. 2022;9:32-8. DOI: 10.1038/s41407-022-0804-3
13. Melo P, Afonso A, Monteiro L, Lopes O, Alves RC. COVID-19 Management in Clinical Dental Care Part II: Personal Protective Equipment for the Dental Care Professional. Int Dent J. 2021 Jun;71(3):263-70. DOI: 10.1016/j.identj.2021.01.007

Attachment to: Schwarz KM, Nienhaus A, Diel R. Risk of SARS-CoV-2 infection in dental healthcare workers – a systematic review and meta-analysis. GMS Hyg Infect Control. 2024;19:Doc09. DOI: 10.3205/dgkh000464

14. Bitencourt FV, Lia EN, Pauletto P, Martins CC, Stefani CM, Massignan C, Canto GL. Prevalence of SARS-CoV-2 infection among oral health care workers worldwide: A meta-analysis. Community Dent Oral Epidemiol. 2023 Oct;51(5):718-28. DOI: 10.1111/cdoe.12827
15. Moher D, Liberati A, Tetzlaff J, Altman DG; PRISMA Group. Preferred reporting items for systematic reviews and meta-analyses: the PRISMA statement. BMJ. 2009 Jul;339:b2535. DOI: 10.1136/bmj.b2535
16. The World Bank. How does the World Bank classify countries? [accessed 2023 Nov 01]. Available from: https://datahelpdesk.worldbank.org/knowledgebase/articles/378834-how-does-the-world-bank-classify-countries
17. Munn Z, Moola S, Lisy K, Riitano D, Tufanaru C. Methodological guidance for systematic reviews of observational epidemiological studies reporting prevalence and cumulative incidence data. Int J Evid Based Healthc. 2015 Sep;13(3):147-53. DOI: 10.1097/XEB.0000000000000054
18. Munn Z, Moola S, Lisy K, Riitano D, Tufanaru C. Chapter 5: Systematic reviews of prevalence and incidence. In: Aromataris E, Munn Z, editors. JBI Manual for Evidence Synthesis. JBI; 2020 [accessed 2023 Oct 23]. DOI: 10.46658/JBIMES-20-06
19. De Sola H, Dueñas M, Salazar A, Ortega-Jiménez P, Failde I. Prevalence of Therapeutic use of Opioids in Chronic non-Cancer Pain Patients and Associated Factors: A Systematic Review and Meta-Analysis. Front Pharmacol. 2020;11:564412. DOI: 10.3389/fphar.2020.564412
20. Abo-Leyah H, Gallant S, Cassidy D, Giam YH, Killick J, Marshall B, Hay G, Snowdon C, Hothersall EJ, Pembridge T, Strachan R, Gallant N, Parcell BJ, George J, Furrie E, Chalmers JD. The protective effect of SARS-CoV-2 antibodies in Scottish healthcare workers. ERJ Open Res. 2021 Apr;7(2). DOI: 10.1183/23120541.00080-2021
21. Abu-Hammad O, Alnazzawi A, Babkair H, Jambi S, Mirah M, Abdouh I, Aljohani RS, Ayeq R, Ghazi L, Al-Subhi H, Dar-Odeh N. COVID-19 Infection in Academic Dental Hospital Personnel; A Cross-Sectional Survey in Saudi Arabia. Int J Environ Res Public Health. 2021 Oct;18(20). DOI: 10.3390/ijerph182010911
22. Akbari N, Salehiniya H, Abbaszadeh H. The prevalence of COVID-19 in dentists and dental assistants. J Biostat Epidemiol. 2021; 7:174-84. DOI: 10.18502/jbe.v7i2.6726
23. Al-Kuwari MG, AbdulMalik MA, Al-Nuaimi AA, Abdulmajeed J, Al-Romaihi HE, Semaan S, Kandy M. Epidemiology Characteristics of COVID-19 Infection Amongst Primary Health Care Workers in Qatar: March-October 2020. Front Public Health. 2021;9:679254. DOI: 10.3389/fpubh.2021.679254
24. Antonio-Villa NE, Bello-Chavolla OY, Vargas-Vázquez A, Fermín-Martínez CA, Márquez-Salinas A, Pisanty-Alatorre J, Bahena-López JP. Assessing the Burden of Coronavirus Disease 2019 (COVID-19) Among Healthcare Workers in Mexico City: A Data-Driven Call to Action. Clin Infect Dis. 2021 Jul;73(1):e191-e198. DOI: 10.1093/cid/ciaa1487
25. Araujo MWB, Estrich CG, Mikkelsen M, Morrissey R, Harrison B, Geisinger ML, Ioannidou E, Vujicic M. COVID-2019 among dentists in the United States: A 6-month longitudinal report of accumulative prevalence and incidence. J Am Dent Assoc. 2021 Jun;152(6):425-33. DOI: 10.1016/j.adaj.2021.03.021
26. Bontà G, Campus G, Cagetti MG. COVID-19 pandemic and dental hygienists in Italy: a questionnaire survey. BMC Health Serv Res. 2020 Oct;20(1):994. DOI: 10.1186/s12913-020-05842-x
27. Cintora P, Rojo R, Martínez A, Ruíz B, Aragoneses JM. Seroprevalence of SARS-CoV-2 in a fully operative dentistry academic center in Madrid (Spain) during the de-escalation phase of the COVID-19 pandemic. Are our dentists at greater risk? Oral Health Prev Dent. 2022; 20:349-53.
28. Duś-Ilnicka I, Szczygielska A, Kuźniarski A, Szymczak A, Pawlik-Sobecka L, Radwan-Oczko M. SARS-CoV-2 IgG Amongst Dental Workers During the COVID-19 Pandemic. Int Dent J. 2022 Jun;72(3):353-9. DOI: 10.1016/j.identj.2022.02.003
29. Estrich CG, Gurenlian JR, Battrell A, Bessner SK, Lynch A, Mikkelsen M, et al. COVID-19 prevalence and related practices among dental hygienists in the United States. J Dent Hyg. 2021; 95:6-16.
30. Ferreira RC, Gomes VE, Rocha NBD, Rodrigues LG, Amaral JHLD, Senna MIB, Alencar GP. COVID-19 Morbidity Among Oral Health Professionals in Brazil. Int Dent J. 2022 Apr;72(2):223-9. DOI: 10.1016/j.identj.2021.05.005

31. Fredriksson L, Cederlund A, Murray M, Jansson L, Skott P. Prevalence of ongoing or previous SARS-CoV-2 infection among dental personnel - the Swedish experience. Acta Odontol Scand. 2023 Mar;81(2):119-23. DOI: 10.1080/00016357.2022.2095023
32. Froum SH, Froum SJ. Incidence of COVID-19 Virus Transmission in Three Dental Offices: A 6-Month Retrospective Study. Int J Periodontics Restorative Dent. 2020;40(6):853-9. DOI: 10.11607/prd.5455
33. Gallus S, Paroni L, Re D, Aiuto R, Battaglia DM, Crippa R, Carugo N, Beretta M, Balsano L, Paglia L. SARS-CoV-2 Infection among the Dental Staff from Lombardy Region, Italy. Int J Environ Res Public Health. 2021 Apr;18(7). DOI: 10.3390/ijerph18073711
34. Hosoglu S, Mahmood MK. COVID-19 infection among dentists in Iraqi Kurdistan Region. J Infect Dev Ctries. 2022 Sep;16(9):1439-44. DOI: 10.3855/jidc.15962
35. Jungo S, Moreau N, Mazevet ME, Ejeil AL, Biosse Duplan M, Salmon B, Smail-Faugeron V. Prevalence and risk indicators of first-wave COVID-19 among oral health-care workers: A French epidemiological survey. PLoS One. 2021;16(2):e0246586. DOI: 10.1371/journal.pone.0246586
36. Lucaciu O, Boca A, Mesaros AS, Petrescu N, Aghiorghiesei O, Mirica IC, Hosu I, Armencea G, Bran S, Dinu CM. Assessing SARS-CoV-2 Infection Rate among Romanian Dental Practitioners. Int J Environ Res Public Health. 2021 May;18(9). DOI: 10.3390/ijerph18094897
37. Madathil S, Siqueira WL, Marin LM, Sanaulla FB, Faraj N, Quiñonez CR, McNally M, Glogauer M, Allison P. The incidence of COVID-19 among dentists practicing in the community in Canada: A prospective cohort study over a 6-month period. J Am Dent Assoc. 2022 May;153(5):450-9.e1. DOI: 10.1016/j.adaj.2021.10.006
38. Mksoud M, Ittermann T, Holtfreter B, Söhnel A, Söhnel C, Welk A, Ulm L, Becker K, Hübner NO, Rau A, Kindler S, Kocher T. Prevalence of SARS-CoV-2 IgG antibodies among dental teams in Germany. Clin Oral Investig. 2022 May;26(5):3965-74. DOI: 10.1007/s00784-021-04363-z
39. Molvik M, Danielsen AS, Grøsland M, Telle KE, Kacelnik O, Eriksen-Volle HM. SARS-CoV-2 in health and care staff in Norway, 2020. Tidsskr Nor Laegeforen; 2021. p. 141.
40. Moraes RR, Correa MB, Martins-Filho PR, Lima GS, Demarco FF. COVID-19 incidence, severity, medication use, and vaccination among dentists: survey during the second wave in Brazil. J Appl Oral Sci. 2022;30:e20220016. DOI: 10.1590/1678-7757-2022-0016
41. Ribeiro JAM, Farias SJS, Souza TAC, Stefani CM, Lima AA, Lia EN. SARS-CoV-2 infection among Brazilian dentists: a seroprevalence study. Braz Oral Res. 2022;36:e035. DOI: 10.1590/1807-3107bor-2022.vol36.0035
42. Rock LD, Madathil S, Khanna M, Macdonald LK, Quiñonez C, Glogauer M, et al. COVID-19 incidence and vaccination rates among Canadian dental hygienists. Can J Dent Hyg. 2022; 56:123-30.
43. Santana LADM, Pinho JNA, de Albuquerque HIM, Souza LMA. COVID-19 contamination among maxillofacial surgeons and impact in Brazilian public center. J Stomatol Oral Maxillofac Surg. 2022 Apr;123(2):92-94. DOI: 10.1016/j.jormas.2021.05.001
44. Sarapultseva M, Hu D, Sarapultsev A. SARS-CoV-2 Seropositivity among Dental Staff and the Role of Aspirating Systems. JDR Clin Trans Res. 2021 Apr;6(2):132-8. DOI: 10.1177/2380084421993099
45. Schmidt J, Perina V, Treglerova J, Pilbauerova N, Suchanek J, Smucler R. COVID-19 Prevalence among Czech Dentists. Int J Environ Res Public Health. 2021 Nov;18(23). DOI: 10.3390/ijerph182312488
46. Puia S, Pasart J, Gualtieri A, Somoza F, Melo C, Alessandrelo M, Gatti P, Squassi A, Rodriguez PA. Corrigendum to "Assesment of SARS-CoV-2 infection-in dentists and supporting staff at a university dental hospital in Argentina". Journal of Oral Biology and Craniofacial Research Volume 11, Issue 2 (2021) Pages 169-173. J Oral Biol Craniofac Res. 2021;11(4):659. DOI: 10.1016/j.jobcr.2021.09.013
47. Shields AM, Faustini SE, Kristunas CA, Cook AM, Backhouse C, Dunbar L, Ebanks D, Emmanuel B, Crouch E, Kröger A, Hirschfeld J, Sharma P, Jaffery R, Nowak S, Gee S, Drayson MT, Richter AG, Dietrich T, Chapple ILC. COVID-19: Seroprevalence and Vaccine Responses in UK Dental Care Professionals. J Dent Res. 2021 Oct;100(11):1220-7. DOI: 10.1177/00220345211020270
48. Suarez-Cabello C, Valdivia E, Vergara-Buenaventura A. Clinical-Epidemiological Profile of Dental Professionals Associated with COVID-19 Infection in Southern Peru: A Cross-Sectional Study. Int J Environ Res Public Health. 2022 Dec;20(1). DOI: 10.3390/ijerph20010672

49. Cagetti MG, Cairoli JL, Senna A, Campus G. COVID-19 Outbreak in North Italy: An Overview on Dentistry. A Questionnaire Survey. Int J Environ Res Public Health. 2020 May;17(11). DOI: 10.3390/ijerph17113835
50. World Health Organization Writing GroupBell D, Nicoll A, Fukuda K, Horby P, Monto A, Hayden F, Wylks C, Sanders L, Van Tam J. Non-pharmaceutical interventions for pandemic influenza, international measures. Emerg Infect Dis. 2006 Jan;12(1):81-7. DOI: 10.3201/eid1201.051370
51. Lerche N, Holtfreter S, Walther B, Semmler T, Al'Sholui F, Dancer SJ, Daeschlein G, Hübner NO, Bröker BM, Papke R, Kohlmann T, Baguhl R, Seifert U, Kramer A. Staphylococcus aureus nasal colonization among dental health care workers in Northern Germany (StaphDent study). Int J Med Microbiol. 2021 Aug;311(6):151524. DOI: 10.1016/j.ijmm.2021.151524
52. Brito-Reia VC, da Silva Bastos R, Vieira Vilhena F, Marques Honório H, Marques da Costa Alves L, Frazão P, Sérgio da Silva Santos P. Population-based virucidal phthalocyanine gargling/rinsing protocol to reduce the risk of coronavirus disease-2019: a community trial. GMS Hyg Infect Control. 2022 Dec 6;17:Doc23. DOI: 10.3205/dgkh000426
53. Kramer A, Eggers M, Exner M, Hübner NO, Simon A, Steinmann E, Walger P, Zwicker P. Recommendation of the German Society of Hospital Hygiene (DGKH): Prevention of COVID-19 by virucidal gargling and virucidal nasal spray - updated version April 2022. GMS Hyg Infect Control. 2022 Jul 7;17:Doc13. DOI: 10.3205/dgkh000416
54. Kramer A, Eggers M, Hübner NO, Walger P, Steinmann E, Exner M. Virucidal gargling and virucidal nasal spray. GMS Hyg Infect Control. 2021 Jan 18;16:Doc02. DOI: 10.3205/ dgkh000373
55. Lenharo M. WHO declares end to COVID-19's emergency phase. Nature. 2023 May 5. DOI: 10.1038/d41586-023-01559-z
56. European Centre for Disease Prevention and Control (ECDC). SARS-CoV-2 variants of concern as of 20 October 2023. 2023 [accessed 2023 Oct 23]. Available from: https://www.ecdc.europa.eu/en/covid-19/variants-concern
57. World Health Organization. From emergency response to long-term COVID-19 disease management: sustaining gains made during the COVID-19 pandemic.Geneva: WHO; 2023 May 03.
58. World Health Organization. WHO Coronavirus (COVID-19) Dashboard. [accessed 2023 Oct 26]. Available from: https://covid19.who.int/
59. Lippi G, Mattiuzzi C, Henry BM. Uncontrolled confounding in COVID-19 epidemiology. Diagnosis (Berl). 2023 May;10(2):200-2. DOI: 10.1515/dx-2022-0128
60. Bundesministerium für Gesundheit. Corona-Testverordnung. [accessed 2023 Nov 02]. Available from: https://www.bundesgesundheitsministerium.de/coronavirus/nationale-teststrategie/coronavirus-testver-ordnung.html
61. ECDC-EMA. ECDC-EM statement on updating COVID-19 vaccines composition for new SARS-CoV-2 virus variants. EMA/257222/2023. European Medicines Agency; 2023 Jun 06
62. European Centre for Disease Prevention and Control (ECDC]. Country overview report: week 40 2023. 2023 Oct 25 [accessed 2023 Nov 02]. Available from: https://www.ecdc.europa.eu/en/covid-19/country-overviews
