# Supplementary material for: Risk of SARS-CoV-2 infection in dental healthcare workers – a systematic review and meta-analysis
Source: GMS Hyg Infect Control. 2024 Mar 5;19:Doc09. doi: 10.3205/dgkh000464 (PMC11035909; doi:10.3205/dgkh000464)
Supplement: Table S3: Joanna Briggs Institute (JBI) critical appraisal tool for prevalence studies [file HIC-19-09-s-003.pdf]

Attachment 3

Table S3: Joanna Briggs Institute (JBI) critical appraisal tool for prevalence studies
The checklist includes 9 questions; for each question, a score is assigned basing on the following answer: yes=1 point; no=0 point; unclear=0 point.

Study	Was the sample frame appropriate to address the target population?	Were study participants sampled in an appropriate way?	Was the sample size adequate?	Were the study subjects and the setting described in detail?	Was the data analysis conducted with sufficient coverage of the identified sample?	Were valid methods used for the identification of the condition?	Was the condition measured in a standard, reliable way for all participants?	Was there appropriate statistical analysis?	Was the response rate adequate, and if not, was the low response rate managed appropriately?	Overall
Abo-Leyah H, et al.	1	1	0	0	1	1	1	0	1	6
Abu-Hammad O, et al.	1	1	1	1	1	1	1	1	1	9
Akbari N, et al.	1	1	U	1	1	1	1	1	1	8
Al Kuwari MG, et al.	1	1	1	0	1	1	1	1	1	8
Antonio-Villa NE, et al.	1	1	1	0	1	1	1	U	1	7
Araujo MWB, et al.	1	1	U	1	1	1	1	1	0	7
Bonta G, et al.	1	1	1	0	1	1	1	1	0	7
Cintora P, et al.	1	1	1	1	1	1	1	1	1	9

Study	Was the sample frame appropriate to address the target population?	Were study participants sampled in an appropriate way?	Was the sample size adequate?	Were the study subjects and the setting described in detail?	Was the data analysis conducted with sufficient coverage of the identified sample?	Were valid methods used for the identification of the condition?	Was the condition measured in a standard, reliable way for all participants?	Was there appropriate statistical analysis?	Was the response rate adequate, and if not, was the low response rate managed appropriately?	Overall
Dus-Ilnicka I, et al.	U	1	1	1	1	1	1	1	1	8
Estrich CG, et al.	1	1	1	1	1	1	1	1	0	8
Ferreira RC, et al.	1	1	1	1	1	1	1	0	1	8
Fredriks-son L, et al.	0	1	1	1	1	1	1	1	1	8
Gallus S, et al.	1	1	1	1	1	1	1	1	1	9
Hosoglu S, et al.	1	1	1	1	1	1	1	1	1	9
Jungo S, et al.	0	1	1	1	1	1	1	1	0	7
Lucaciu O, et al.	0	1	1	1	1	1	1	1	0	7
Madathil S, et al.	1	1	1	1	1	1	1	1	1	9

References

1. WHO. Coronavirus disease (COVID-19). Geneva: World Health Organization; 2023 [accessed 2023 Oct 26]. Available from: https://www.who.int/emergencies/diseases/novel-coronavirus-2019
2. Global tuberculosis report 2022. Geneva: World Health organization; 2022. p. 68.
3. Thaweethai T, Jolley SE, Karlson EW, Levitan EB, Levy B, McComsey GA, McCorkell L, Nadkarni GN, Parthasarathy S, Singh U, Walker TA, Selvaggi CA, Shinnick DJ, Schulte CCM, Atchley-Challenner R, Alba GA, Alicic R, Altman N, Anglin K, Argueta U, Ashktorab H, Baslet G, Bassett IV, Bateman L, Bedi B, Bhattacharyya S, Bind MA, Blomkalns AL, Bonilla H, Bush PA, Castro M, Chan J, Charney AW, Chen P, Chibnik LB, Chu HY, Clifton RG, Costantine MM, Cribbs SK, Davila Nieves SI, Deeks SG, Duven A, Emery IF, Erdmann N, Erlandson KM, Ernst KC, Farah-Abraham R, Farner CE, Feuerriegel EM, Fleurimont J, Fonseca V, Franko N, Gainer V, Gander JC, Gardner EM, Geng LN, Gibson KS, Go M, Goldman JD, Grebe H, Greenway FL, Habli M, Hafner J, Han JE, Hanson KA, Heath J, Hernandez C, Hess R, Hodder SL, Hoffman MK, Hoover SE, Huang B, Hughes BL, Jagannathan P, John J, Jordan MR, Katz SD, Kaufman ES, Kelly JD, Kelly SW, Kemp MM, Kirwan JP, Klein JD, Knox KS, Krishnan JA, Kumar A, Laiyemo AO, Lambert AA, Lanca M, Lee-Iannotti JK, Logarbo BP, Longo MT, Luciano CA, Lutrick K, Maley JH, Marathe JG, Marconi V, Marshall GD, Martin CF, Matusov Y, Mehari A, Mendez-Figueroa H, Mermelstein R, Metz TD, Morse R, Mosier J, Mouchati C, Mullington J, Murphy SN, Neuman RB, Nikolich JZ, Ofotokun I, Ojemakinde E, Palatnik A, Palomares K, Parimon T, Parry S, Patterson JE, Patterson TF, Patzer RE, Peluso MJ, Pemu P, Pettker CM, Plunkett BA, Pogreba-Brown K, Poppas A, Quigley JG, Reddy U, Reece R, Reeder H, Reeves WB, Reiman EM, Rischard F, Rosand J, Rouse DJ, Ruff A, Saade G, Sandoval GJ, Schlater SM, Shepherd F, Sherif ZA, Simhan H, Singer NG, Skupski DW, Sowles A, Sparks JA, Sukhera FI, Taylor BS, Teunis L, Thomas RJ, Thorp JM, Thuluvath P, Ticotsky A, Tita AT, Tuttle KR, Urdaneta AE, Valdivieso D, VanWagoner TM, Vasey A, Verduzco-Gutierrez M, Wallace ZS, Ward HD, Warren DE, Weiner SJ, Welch S, Whiteheart SW, Wiley Z, Wisnivesky JP, Yee LM, Zisis S, Horwitz LI, Foulkes AS; RECOVER Consortium. Development of a Definition of Postacute Sequelae of SARS-CoV-2 Infection. JAMA. 2023 Jun;329(22):1934-46. DOI: 10.1001/jama.2023.8823
4. Darwish S, El-Boghdadly K, Edney C, Babbar A, Shembesh T. Respiratory protection in dentistry. Br Dent J. 2021 Feb;230(4):207-14. DOI: 10.1038/s41415-021-2657-0
5. Meethil AP, Saraswat S, Chaudhary PP, Dabdoub SM, Kumar PS. Sources of SARS-CoV-2 and Other Microorganisms in Dental Aerosols. J Dent Res. 2021 Jul;100(8):817-23. DOI: 10.1177/00220345211015948
6. Graziani F, Izzetti R, Lardani L, Totaro M, Baggiani A. Experimental Evaluation of Aerosol Production after Dental Ultrasonic Instrumentation: An Analysis on Fine Particulate Matter Perturbation. Int J Environ Res Public Health. 2021 Mar;18(7). DOI: 10.3390/ijerph18073357
7. Centers for Disease Control and Prevention (CDC). Interim guidance on infection control measures for 2009 H1N1 influenza in healthcare settings, including protection of healthcare personnel. Atlanta, GA: CDC; 2010 Jul 15 [accessed 2023 Oct 27]. Available from: https://www.cdc.gov/h1n1flu/guidelines_infection_control.htm.
8. Lee V, Yap J, Cook AR, Chen M, Tay J, Barr I, Kelso A, Tan B, Loh JP, Lin R, Cui L, Kelly PM, Leo Y, Chia K, Kang WL, Tambyah P, Seet B. Effectiveness of public health measures in mitigating pandemic influenza spread: a prospective sero-epidemiological cohort study. J Infect Dis. 2010 Nov;202(9):1319-26. DOI: 10.1086/656480
9. Marshall C, Kelso A, McBryde E, Barr IG, Eisen DP, Sasadeusz J, Buising K, Cheng AC, Johnson P, Richards M. Pandemic (H1N1) 2009 risk for frontline health care workers. Emerg Infect Dis. 2011 Jun;17(6):1000-6. DOI: 10.3201/eid/1706.101030
10. Yen TY, Lu CY, Chang LY, Tsai YT, Huang LM. Longitudinal seroepidemiologic study of the 2009 pandemic influenza A (H1N1) infection among health care workers in a children's hospital. BMC Infect Dis. 2012 Apr;12:89. DOI: 10.1186/1471-2334-12-89
11. Radonovich LJ Jr, Simberkoff MS, Bessesen MT, Brown AC, Cummings DAT, Gaydos CA, Los JG, Krosche AE, Gibert CL, Gorse GJ, Nyquist AC, Reich NG, Rodriguez-Barradas MC, Price CS, Perl TM; ResPECT investigators. N95 Respirators vs Medical Masks for Preventing Influenza Among Health Care Personnel: A Randomized Clinical Trial. JAMA. 2019 Sep;322(9):824-33. DOI: 10.1001/jama.2019.11645
12. Savage A. An evaluation of the impact of COVID-19 on the leadership behaviour of dental practice managers in England. BDJ Team. 2022;9:32-8. DOI: 10.1038/s41407-022-0804-3
13. Melo P, Afonso A, Monteiro L, Lopes O, Alves RC. COVID-19 Management in Clinical Dental Care Part II: Personal Protective Equipment for the Dental Care Professional. Int Dent J. 2021 Jun;71(3):263-70. DOI: 10.1016/j.identj.2021.01.007

14. Bitencourt FV, Lia EN, Pauletto P, Martins CC, Stefani CM, Massignan C, Canto GL. Prevalence of SARS-CoV-2 infection among oral health care workers worldwide: A meta-analysis. Community Dent Oral Epidemiol. 2023 Oct;51(5):718-28. DOI: 10.1111/cdoe.12827
15. Moher D, Liberati A, Tetzlaff J, Altman DG; PRISMA Group. Preferred reporting items for systematic reviews and meta-analyses: the PRISMA statement. BMJ. 2009 Jul;339:b2535. DOI: 10.1136/bmj.b2535
16. The World Bank. How does the World Bank classify countries? [accessed 2023 Nov 01]. Available from: https://datahelpdesk.worldbank.org/knowledgebase/articles/378834-how-does-the-world-bank-classify-countries
17. Munn Z, Moola S, Lisy K, Riitano D, Tufanaru C. Methodological guidance for systematic reviews of observational epidemiological studies reporting prevalence and cumulative incidence data. Int J Evid Based Healthc. 2015 Sep;13(3):147-53. DOI: 10.1097/XEB.0000000000000054
18. Munn Z, Moola S, Lisy K, Riitano D, Tufanaru C. Chapter 5: Systematic reviews of prevalence and incidence. In: Aromataris E, Munn Z, editors. JBI Manual for Evidence Synthesis. JBI; 2020 [accessed 2023 Oct 23]. DOI: 10.46658/JBIMES-20-06
19. De Sola H, Dueñas M, Salazar A, Ortega-Jiménez P, Failde I. Prevalence of Therapeutic use of Opioids in Chronic non-Cancer Pain Patients and Associated Factors: A Systematic Review and Meta-Analysis. Front Pharmacol. 2020;11:564412. DOI: 10.3389/fphar.2020.564412
20. Abo-Leyah H, Gallant S, Cassidy D, Giam YH, Killick J, Marshall B, Hay G, Snowdon C, Hothersall EJ, Pembridge T, Strachan R, Gallant N, Parcell BJ, George J, Furrie E, Chalmers JD. The protective effect of SARS-CoV-2 antibodies in Scottish healthcare workers. ERJ Open Res. 2021 Apr;7(2). DOI: 10.1183/23120541.00080-2021
21. Abu-Hammad O, Alnazzawi A, Babkair H, Jambi S, Mirah M, Abdouh I, Aljohani RS, Ayeq R, Ghazi L, Al-Subhi H, Dar-Odeh N. COVID-19 Infection in Academic Dental Hospital Personnel; A Cross-Sectional Survey in Saudi Arabia. Int J Environ Res Public Health. 2021 Oct;18(20). DOI: 10.3390/ijerph182010911
22. Akbari N, Salehiniya H, Abbaszadeh H. The prevalence of COVID-19 in dentists and dental assistants. J Biostat Epidemiol. 2021; 7:174-84. DOI: 10.18502/jbe.v7i2.6726
23. Al-Kuwari MG, AbdulMalik MA, Al-Nuaimi AA, Abdulmajeed J, Al-Romaihi HE, Semaan S, Kandy M. Epidemiology Characteristics of COVID-19 Infection Amongst Primary Health Care Workers in Qatar: March-October 2020. Front Public Health. 2021;9:679254. DOI: 10.3389/fpubh.2021.679254
24. Antonio-Villa NE, Bello-Chavolla OY, Vargas-Vázquez A, Fermín-Martínez CA, Márquez-Salinas A, Pisanty-Alatorre J, Bahena-López JP. Assessing the Burden of Coronavirus Disease 2019 (COVID-19) Among Healthcare Workers in Mexico City: A Data-Driven Call to Action. Clin Infect Dis. 2021 Jul;73(1):e191-e198. DOI: 10.1093/cid/ciaa1487
25. Araujo MWB, Estrich CG, Mikkelsen M, Morrissey R, Harrison B, Geisinger ML, Ioannidou E, Vujicic M. COVID-2019 among dentists in the United States: A 6-month longitudinal report of accumulative prevalence and incidence. J Am Dent Assoc. 2021 Jun;152(6):425-33. DOI: 10.1016/j.adaj.2021.03.021
26. Bontà G, Campus G, Cagetti MG. COVID-19 pandemic and dental hygienists in Italy: a questionnaire survey. BMC Health Serv Res. 2020 Oct;20(1):994. DOI: 10.1186/s12913-020-05842-x
27. Cintora P, Rojo R, Martínez A, Ruíz B, Aragoneses JM. Seroprevalence of SARS-CoV-2 in a fully operative dentistry academic center in Madrid (Spain) during the de-escalation phase of the COVID-19 pandemic. Are our dentists at greater risk? Oral Health Prev Dent. 2022; 20:349-53.
28. Duś-Ilnicka I, Szczygielska A, Kuźniarski A, Szymczak A, Pawlik-Sobecka L, Radwan-Oczko M. SARS-CoV-2 IgG Amongst Dental Workers During the COVID-19 Pandemic. Int Dent J. 2022 Jun;72(3):353-9. DOI: 10.1016/j.identj.2022.02.003
29. Estrich CG, Gurenlian JR, Battrell A, Bessner SK, Lynch A, Mikkelsen M, et al. COVID-19 prevalence and related practices among dental hygienists in the United States. J Dent Hyg. 2021; 95:6-16.
30. Ferreira RC, Gomes VE, Rocha NBD, Rodrigues LG, Amaral JHLD, Senna MIB, Alencar GP. COVID-19 Morbidity Among Oral Health Professionals in Brazil. Int Dent J. 2022 Apr;72(2):223-9. DOI: 10.1016/j.identj.2021.05.005
31. Fredriksson L, Cederlund A, Murray M, Jansson L, Skott P. Prevalence of ongoing or previous SARS-CoV-2 infection among dental personnel - the Swedish experience. Acta Odontol Scand. 2023 Mar;81(2):119-23. DOI: 10.1080/00016357.2022.2095023

32. Froum SH, Froum SJ. Incidence of COVID-19 Virus Transmission in Three Dental Offices: A 6-Month Retrospective Study. Int J Periodontics Restorative Dent. 2020;40(6):853-9. DOI: 10.11607/prd.5455
33. Gallus S, Paroni L, Re D, Aiuto R, Battaglia DM, Crippa R, Carugo N, Beretta M, Balsano L, Paglia L. SARS-CoV-2 Infection among the Dental Staff from Lombardy Region, Italy. Int J Environ Res Public Health. 2021 Apr;18(7). DOI: 10.3390/ijerph18073711
34. Hosoglu S, Mahmood MK. COVID-19 infection among dentists in Iraqi Kurdistan Region. J Infect Dev Ctries. 2022 Sep;16(9):1439-44. DOI: 10.3855/jidc.15962
35. Jungo S, Moreau N, Mazevet ME, Ejeil AL, Biosse Duplan M, Salmon B, Smail-Faugeron V. Prevalence and risk indicators of first-wave COVID-19 among oral health-care workers: A French epidemiological survey. PLoS One. 2021;16(2):e0246586. DOI: 10.1371/journal.pone.0246586
36. Lucaciu O, Boca A, Mesaros AS, Petrescu N, Aghiorghiesei O, Mirica IC, Hosu I, Armencea G, Bran S, Dinu CM. Assessing SARS-CoV-2 Infection Rate among Romanian Dental Practitioners. Int J Environ Res Public Health. 2021 May;18(9). DOI: 10.3390/ijerph18094897
37. Madathil S, Siqueira WL, Marin LM, Sanaulla FB, Faraj N, Quiñonez CR, McNally M, Glogauer M, Allison P. The incidence of COVID-19 among dentists practicing in the community in Canada: A prospective cohort study over a 6-month period. J Am Dent Assoc. 2022 May;153(5):450-9.e1. DOI: 10.1016/j.adaj.2021.10.006
38. Mksoud M, Ittermann T, Holtfreter B, Söhnel A, Söhnel C, Welk A, Ulm L, Becker K, Hübner NO, Rau A, Kindler S, Kocher T. Prevalence of SARS-CoV-2 IgG antibodies among dental teams in Germany. Clin Oral Investig. 2022 May;26(5):3965-74. DOI: 10.1007/s00784-021-04363-z
39. Molvik M, Danielsen AS, Grøsland M, Telle KE, Kacelnik O, Eriksen-Volle HM. SARS-CoV-2 in health and care staff in Norway, 2020. Tidsskr Nor Laegeforen; 2021. p. 141.
40. Moraes RR, Correa MB, Martins-Filho PR, Lima GS, Demarco FF. COVID-19 incidence, severity, medication use, and vaccination among dentists: survey during the second wave in Brazil. J Appl Oral Sci. 2022;30:e20220016. DOI: 10.1590/1678-7757-2022-0016
41. Ribeiro JAM, Farias SJS, Souza TAC, Stefani CM, Lima AA, Lia EN. SARS-CoV-2 infection among Brazilian dentists: a seroprevalence study. Braz Oral Res. 2022;36:e035. DOI: 10.1590/1807-3107bor-2022.vol36.0035
42. Rock LD, Madathil S, Khanna M, Macdonald LK, Quiñonez C, Glogauer M, et al. COVID-19 incidence and vaccination rates among Canadian dental hygienists. Can J Dent Hyg. 2022; 56:123-30.
43. Santana LADM, Pinho JNA, de Albuquerque HIM, Souza LMA. COVID-19 contamination among maxillofacial surgeons and impact in Brazilian public center. J Stomatol Oral Maxillofac Surg. 2022 Apr;123(2):92-94. DOI: 10.1016/j.jormas.2021.05.001
44. Sarapultseva M, Hu D, Sarapultsev A. SARS-CoV-2 Seropositivity among Dental Staff and the Role of Aspirating Systems. JDR Clin Trans Res. 2021 Apr;6(2):132-8. DOI: 10.1177/2380084421993099
45. Schmidt J, Perina V, Treglerova J, Pilbauerova N, Suchanek J, Smucler R. COVID-19 Prevalence among Czech Dentists. Int J Environ Res Public Health. 2021 Nov;18(23). DOI: 10.3390/ijerph182312488
46. Puia S, Pasart J, Gualtieri A, Somoza F, Melo C, Alessandrelo M, Gatti P, Squassi A, Rodriguez PA. Corrigendum to “Assesment of SARS-CoV-2 infection-in dentists and supporting staff at a university dental hospital in Argentina”. Journal of Oral Biology and Craniofacial Research Volume 11, Issue 2 (2021) Pages 169-173. J Oral Biol Craniofac Res. 2021;11(4):659. DOI: 10.1016/j.jobcr.2021.09.013
47. Shields AM, Faustini SE, Kristunas CA, Cook AM, Backhouse C, Dunbar L, Ebanks D, Emmanuel B, Crouch E, Kröger A, Hirschfeld J, Sharma P, Jaffery R, Nowak S, Gee S, Drayson MT, Richter AG, Dietrich T, Chapple ILC. COVID-19: Seroprevalence and Vaccine Responses in UK Dental Care Professionals. J Dent Res. 2021 Oct;100(11):1220-7. DOI: 10.1177/00220345211020270
48. Suarez-Cabello C, Valdivia E, Vergara-Buenaventura A. Clinical-Epidemiological Profile of Dental Professionals Associated with COVID-19 Infection in Southern Peru: A Cross-Sectional Study. Int J Environ Res Public Health. 2022 Dec;20(1). DOI: 10.3390/ijerph20010672
49. Cagetti MG, Cairoli JL, Senna A, Campus G. COVID-19 Outbreak in North Italy: An Overview on Dentistry. A Questionnaire Survey. Int J Environ Res Public Health. 2020 May;17(11). DOI: 10.3390/ijerph17113835

50. World Health Organization Writing GroupBell D, Nicoll A, Fukuda K, Horby P, Monto A, Hayden F, Wylks C, Sanders L, Van Tam J. Non-pharmaceutical interventions for pandemic influenza, international measures. Emerg Infect Dis. 2006 Jan;12(1):81-7. DOI: 10.3201/eid1201.051370
51. Lerche N, Holtfreter S, Walther B, Semmler T, Al'Sholui F, Dancer SJ, Daeschlein G, Hübner NO, Bröker BM, Papke R, Kohlmann T, Baguhl R, Seifert U, Kramer A. Staphylococcus aureus nasal colonization among dental health care workers in Northern Germany (StaphDent study). Int J Med Microbiol. 2021 Aug;311(6):151524. DOI: 10.1016/j.ijmm.2021.151524
52. Brito-Reia VC, da Silva Bastos R, Vieira Vilhena F, Marques Honório H, Marques da Costa Alves L, Frazão P, Sérgio da Silva Santos P. Population-based virucidal phthalocyanine gargling/rinsing protocol to reduce the risk of coronavirus disease-2019: a community trial. GMS Hyg Infect Control. 2022 Dec 6;17:Doc23. DOI: 10.3205/dgkh000426
53. Kramer A, Eggers M, Exner M, Hübner NO, Simon A, Steinmann E, Walger P, Zwicker P. Recommendation of the German Society of Hospital Hygiene (DGKH): Prevention of COVID-19 by virucidal gargling and virucidal nasal spray - updated version April 2022. GMS Hyg Infect Control. 2022 Jul 7;17:Doc13. DOI: 10.3205/dgkh000416
54. Kramer A, Eggers M, Hübner NO, Walger P, Steinmann E, Exner M. Virucidal gargling and virucidal nasal spray. GMS Hyg Infect Control. 2021 Jan 18;16:Doc02. DOI: 10.3205/ dgkh000373
55. Lenharo M. WHO declares end to COVID-19's emergency phase. Nature. 2023 May 5. DOI: 10.1038/d41586-023-01559-z
56. European Centre for Disease Prevention and Control (ECDC). SARS-CoV-2 variants of concern as of 20 October 2023. 2023 [accessed 2023 Oct 23]. Available from: https://www.ecdc.europa.eu/en/covid-19/variants-concern
57. World Health Organization. From emergency response to long-term COVID-19 disease management: sustaining gains made during the COVID-19 pandemic.Geneva: WHO; 2023 May 03.
58. World Health Organization. WHO Coronavirus (COVID-19) Dashboard. [accessed 2023 Oct 26]. Available from: https://covid19.who.int/
59. Lippi G, Mattiuzzi C, Henry BM. Uncontrolled confounding in COVID-19 epidemiology. Diagnosis (Berl). 2023 May;10(2):200-2. DOI: 10.1515/dx-2022-0128
60. Bundesministerium für Gesundheit. Corona-Testverordnung. [accessed 2023 Nov 02]. Available from: https://www.bundesgesundheitsministerium.de/coronavirus/nationale-teststrategie/coronavirus-testver-ordnung.html
61. ECDC-EMA. ECDC-EM statement on updating COVID-19 vaccines composition for new SARS-CoV-2 virus variants. EMA/257222/2023. European Medicines Agency; 2023 Jun 06
62. European Centre for Disease Prevention and Control (ECDC]. Country overview report: week 40 2023. 2023 Oct 25 [accessed 2023 Nov 02]. Available from: https://www.ecdc.europa.eu/en/covid-19/country-overviews
